# NMR Studies of Retroviral Genome Packaging

**DOI:** 10.3390/v12101115

**Published:** 2020-09-30

**Authors:** Patricia S. Boyd, Janae B. Brown, Joshua D. Brown, Jonathan Catazaro, Issac Chaudry, Pengfei Ding, Xinmei Dong, Jan Marchant, Colin T. O’Hern, Karndeep Singh, Canessa Swanson, Michael F. Summers, Saif Yasin

**Affiliations:** Howard Hughes Medical Institute, Department of Chemistry and Biochemistry, University of Maryland Baltimore County, Baltimore, MD 21250, USA; pboyd2@umbc.edu (P.S.B.); janaeb@umbc.edu (J.B.B.); jdbrown@umbc.edu (J.D.B.); jcataz@umbc.edu (J.C.); ichaudr1@umbc.edu (I.C.); dingp@umbc.edu (P.D.); dx1@umbc.edu (X.D.); janm@umbc.edu (J.M.); cohern1@umbc.edu (C.T.O.); ksingh5@umbc.edu (K.S.); canessa@umbc.edu (C.S.); saif.yasin@som.umaryland.edu (S.Y.)

**Keywords:** retrovirus, genome, packaging, NMR, cyro-EM, SAXS, RNA, protein, structure

## Abstract

Nearly all retroviruses selectively package two copies of their unspliced RNA genomes from a cellular milieu that contains a substantial excess of non-viral and spliced viral RNAs. Over the past four decades, combinations of genetic experiments, phylogenetic analyses, nucleotide accessibility mapping, in silico RNA structure predictions, and biophysical experiments were employed to understand how retroviral genomes are selected for packaging. Genetic studies provided early clues regarding the protein and RNA elements required for packaging, and nucleotide accessibility mapping experiments provided insights into the secondary structures of functionally important elements in the genome. Three-dimensional structural determinants of packaging were primarily derived by nuclear magnetic resonance (NMR) spectroscopy. A key advantage of NMR, relative to other methods for determining biomolecular structure (such as X-ray crystallography), is that it is well suited for studies of conformationally dynamic and heterogeneous systems—a hallmark of the retrovirus packaging machinery. Here, we review advances in understanding of the structures, dynamics, and interactions of the proteins and RNA elements involved in retroviral genome selection and packaging that are facilitated by NMR.

## 1. Introduction

**Figure 1:**
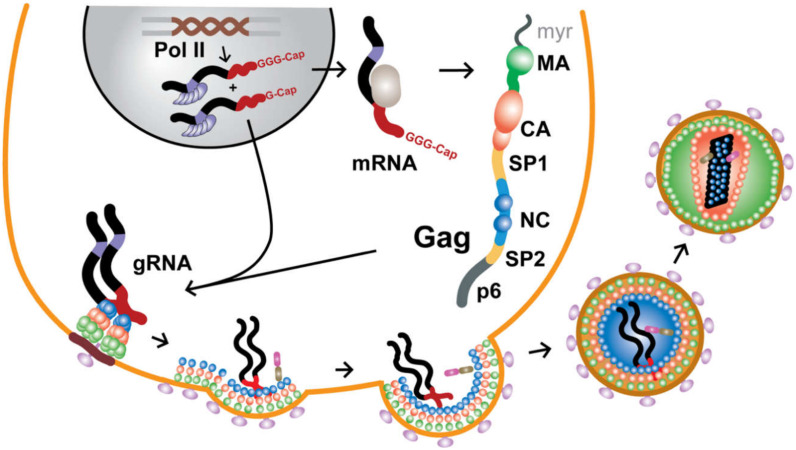
Late phase of the HIV-1 replication cycle. Heterogeneous transcriptional start site usage affords 5′-capped transcripts beginning with a single guanosine that function as genomes (gRNA) and are packaged as dimers, as well as 5′-capped transcripts beginning with two (minor species, not shown) or three guanosines that function as mRNAs (5ʹ-leader, coding region, and Rev-binding sites of the viral RNA shown in black, red, and gray, respectively).

During the late phase of the retroviral replication cycle (after the viral genome is reverse transcribed and the proviral DNA is integrated into the genome of the host cell), two copies of the unspliced, 5′-capped, and 3′-polyadenylated RNA transcript are specifically selected for packaging into assembling virions. Genomes are selected against a background that includes a substantial excess of non-genomic RNA, including cellular mRNAs [1], monomeric unspliced viral transcripts that encode for the viral Gag and Gag-Pol polyproteins, and spliced viral mRNAs that encode for accessory and envelope proteins—all of which are excluded during virus assembly. Over the past ~40 years, considerable effort was made to understand the mechanisms through which retroviruses achieve such exquisite selectivity (for reviews, see [1,2,3,4,5,6,7,8,9,10,11,12,13,14,15,16,17]). RNA packaging and virus assembly are both mediated by the Gag polyproteins, which contain three independently folded domains [matrix (MA), capsid (CA), and nucleocapsid (NC)] and additional domains that are intrinsically disordered (for HIV-1, these are: SP1, SP2, p6), Figure 1. The MA domain functions in the selective targeting of Gag to virus assembly sites on the plasma membrane, the CA domain promotes Gag self-association, and the NC domain recognizes and binds the cognate RNA genome. In addition, the conformationally dynamic SP1 domain [18] that bridges the CA and NC domains, adopts a 6-helix bundle structure that stabilizes Gag:Gag interactions in immature virus particles [19,20,21,22]. During or shortly after budding, retroviruses undergo a maturation process in which these domains are liberated by proteolytic cleavage of Gag, leading to large changes in viral morphology; Figure 1. The liberated CA proteins condense to form a conical capsid core particle that encapsidates the viral genome, along with the essential enzymes needed for reverse transcription and subsequent integration of the proviral DNA.

The *Retroviridae* family includes two subfamilies encompassing eleven genera, categorized primarily by sequence similarity within the *pol* gene [23]. The *Spumaretrovirinae* subfamily (*Bovispumavirus*, *Equispumavirus*, *Felispumavirus*, *Prosimiispumavirus*, *Simiispumavirus*) diverged early and is evolutionarily distant from the *Orthoretrovirinae* subfamily (*Alpharetrovirus*, *Betaretrovirus*, *Deltaretrovirus*, *Epsilonretrovirus*, *Gammaretrovirus*, *Lentivirus*). Evolutionarily related retroviruses within a given genus can preferentially package each other’s viral genomes [24,25,26], and the packaging assays indicate that this property is conserved within *lentiviruses* (HIV and simian immunodeficiency virus (SIV)) [24], as well as *gammaretroviruses* (Moloney murine sarcoma virus and spleen necrosis virus) [25]. Retroviruses in distinct genera are also found to maintain this property. HIV-1 chimeric virions that contain the Moloney murine leukemia virus (MoMuLV) NC domain, preferentially package the MoMuLV RNA [27], whereas the MoMuLV chimeric virions that contain the HIV-1 NC domain, package the HIV-1 genome [28], indicating that it is the NC domain of Gag that is responsible for genome selection. In fact, the MoMuLV chimeric virions packaged the full-length unspliced HIV-1 genome, indicating that chimeric precursor Gag proteins containing the HIV-1 NC domain can also distinguish between unspliced and spliced viral RNAs, during genome selection [28]. Although it is unclear where in the cell NC–RNA interactions initially occur, confocal studies showed that virus assembly is nucleated by the binding of a small number of Gag proteins (possibly a dozen or fewer [29]) to the viral genome, at plasma membrane assembly sites [30], which leads to the subsequent recruitment of several thousand additional Gag proteins [29,31] and cellular factors that promote virus budding [32,33]. Although virions can assemble in the absence of their genomes by incorporating an equivalent amount of cellular RNAs [34,35,36,37,38,39], vector RNAs containing the authentic packaging signal can efficiently out-compete these more abundant RNAs for packaging [40,41], as long as the non-native downstream vector residues do not interfere with proper folding of the packaging signal [42].

Retroviruses contain two copies of their genomes [43], both of which are utilized for strand transfer-mediated recombination during reverse transcription [44,45,46]. As only one DNA allele is generated, retroviruses are considered “pseudodiploid.” In virions, the genomes form non-covalently linked dimers that become increasingly stable towards thermal denaturation, with increasing virus age [47,48,49,50,51,52]. Dimerization might occur co-transcriptionally [53] but most likely after the genomes are exported from the nucleus [54] and possibly not until they reach the plasma membrane [30]. Dimerization, packaging, and other RNA-dependent functions (including transcriptional activation, splicing, and initiation of reverse transcription) are promoted by the elements located within the 5′-leader of the RNA [1,2,3,4,5,6,7,8,9,10,11,12,13,14,16,17], which is among the most conserved regions of the genome [55,56], (http://www.hiv.lanl.gov/). Although some studies with mutant genomes suggested that residues in the *gag* open reading frame might also be important for packaging, it now appears that packaging defects were caused by misfolding of the 5′-leader due to interactions with non-native downstream sequences [42]. Studies identified regions within the 5′-leaders of HIV-1 [40], MoMuLV [57,58], and Rous Sarcoma Virus (RSV) [59,60,61,62,63,64], which are independently capable of directing heterologous RNAs into assembling virus-like particles (VLPs). These “core encapsidation signals” incorporate or reside near residues that promote RNA dimerization [3,4,6,8] and there is considerable evidence that genome packaging is critically dependent on dimerization [1,2,11,65,66,67,68]. Although dimerization could be modulated by a riboswitch-like mechanism promoted by the chaperone activity of NC [69,70,71], recent studies indicate that RNA fates are instead controlled at the level of transcription, through the heterogeneous start site usage [72,73,74,75]. In this mechanism, 5′-capped RNAs transcribed with a single 5′-guanosine preferentially form dimers that are packaged into virions, where they function as genomes (gRNA). In contrast, 5′-capped transcripts that begin with two or three guanosines form monomers that promote splicing, are retained in cells, and function as mRNAs [72,73,74,75]; Figure 1.

Solution-state nuclear magnetic resonance (NMR) contributed substantially to current knowledge of the structures and mechanisms that contribute to retroviral genome selection and packaging. NMR offers a number of advantages for structural characterization of biomolecules, including the ability to characterize structures and dynamics of conformationally heterogeneous samples, and to obtain data without the need to prepare crystalline samples. Due to these advantages, seven of the first twelve HIV-1 protein domain structures that were solved during the first decade of HIV-1 structural biology were determined by NMR [76]. However, NMR also has a number of technical disadvantages that confound the studies of the RNA components that are important for genome packaging. Since most RNAs contain only four types of ribonucleotides (guanosine, G; cytidine, C; adenosine, A; uridine, U), chemical shift dispersion is relatively low. Proton density is also lower than that of proteins, particularly for non-exchangeable protons that are found mainly within the major grooves of A-form helices. Interproton distances between different secondary structure elements are typically greater than 5 Å [77], which limits the utility of nuclear Overhauser effect (NOE) experiments for establishing the overall RNA folds [78,79]. In addition, H–C dipolar coupling can severely limit the sensitivity and resolution of the H–C correlation NMR spectra obtainable for larger RNAs with longer rotational correlation times. For these reasons, high-resolution NMR-based structural studies were generally limited to relatively small RNAs [80,81]. The average size of NMR-derived RNA structures deposited in public databases is 30 nucleotides and only six structures were reported for RNAs comprising more than 100 nucleotides (http://www.rcsb.org). However, new ^2^H-edited NMR methods recently enabled structural studies of RNA elements within the intact, dimeric 5′-leaders of two strains of HIV-1 (HIV-1_NL4-3_ and HIV-1_MAL_) (>700 nucleotide dimers; >230 kDa) [71,75,81]. Hybrid approaches that combine high-resolution local structural information provided by NMR with lower-resolution global structural information from cryogenic electron microscopy (cryo-EM), or small angle X-ray scattering (SAXS) could enable structural studies of even larger RNAs and protein–RNA complexes that are important for packaging. Here, we review how NMR studies contributed to the current understanding of the structural and mechanistic determinants of retroviral genome selection and packaging.

## 2. Protein Components Important for Genome Packaging

### 2.1. Nucleocapsid Domain of Gag

**Figure 2:**
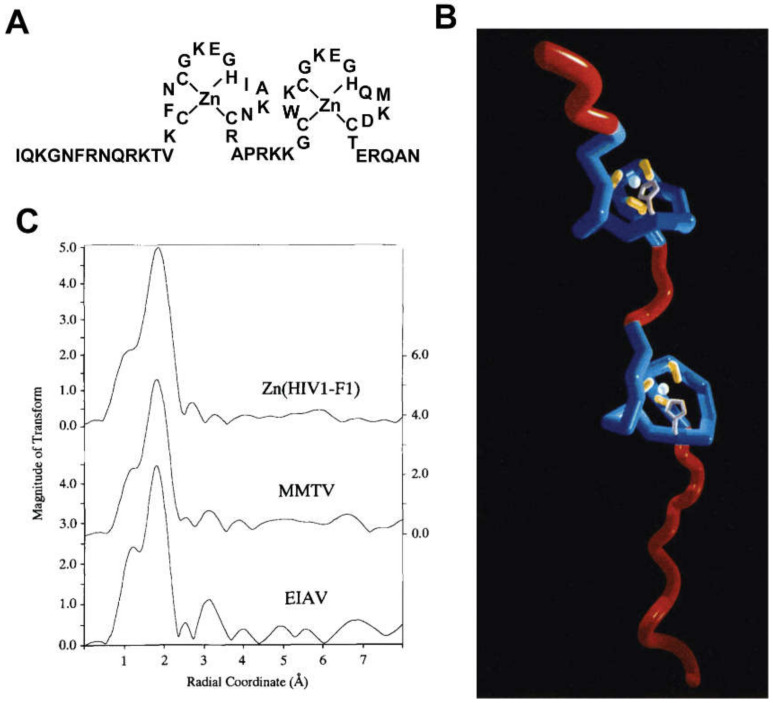
HIV-1 NC is a zinc metalloprotein. (**A**) Amino acid sequence and zinc binding mode of the HIV-1_NL4-3_ NC protein. (**B**) NMR structure of HIV-1_NL4-3_ NC. The independently folded CCHC “zinc knuckle” domains behave like “beads on a string.” (**C**) Similarities of zinc-edge EXAFS spectra obtained for an isolated HIV-1 CCHC peptide (top) and for intact retroviruses [Mouse Mammary Tumor Virus (center) and Equine Infectious Anemia Virus (bottom)]. Panels (**B**,**C**) reproduced from [82], with permission.

The NC domain of the HIV-1 Gag protein was the first retroviral constituent studied by NMR. Except for the spumaviruses, all retroviral NC proteins contain one or two copies of a conserved cysteine/histidine rich sequence (CCHC; C = cysteine, H = histidine) [82] that was originally discovered by Henderson et al. [83]. After the subsequent discovery of CCHH arrays in *Xenopus* protein transcription factors and proposals that these arrays function as nucleic acid-binding “zinc fingers” [84], Berg et al. proposed that the retroviral CCHC arrays might also function as zinc binding sites [85]; Figure 2A. Although this proposal was initially controversial [86,87], mutagenesis studies showed that the substitution of a single Cys by Ser (i.e., a single atom S to O substitution) completely ablated viral replication [88]. Furthermore, Goff et al. showed that mutation of several conserved (or conservatively substituted) hydrophobic residues within the CCHC arrays can significantly reduce RNA packaging specificity [89]. Berg et al. showed that an 18-residue CCHC array is capable of binding both Co^2+^ and Zn^2+^ with high affinity, likely via 3S/1N coordination [90]. ^1^H-^113^Cd heteronuclear spin echo difference NMR applied to a ^113^Cd-substituted HIV-1 CCHC peptide, revealed that the cysteine-S and histidine-Nε atoms were coordinated to the metal [91], and NOE-based structural studies showed that the peptide adopts a mini-globular “zinc knuckle” structure, upon coordination of zinc [92]. Subsequent NMR studies showed that the CCHC arrays of virus-isolated NC form independently folded zinc knuckle domains that behave like “beads on a string” [93]; Figure 2B. Zinc-edge extended X-ray absorption fine structure spectroscopy confirmed that the CCHC arrays are populated with zinc in mature particles [94]; Figure 2C.

NMR structures were reported for several isolated retroviral zinc knuckle domains [92,93,95,96,97,98,99], for intact NC proteins [82,100,101,102,103,104,105,106], and NC–RNA complexes [66,104,107,108,109,110,111,112,113,114] (see sections below for more details). Weak NOEs between aromatic residues of the N- and C-terminal HIV-1 NC zinc knuckles [102] and NMR relaxation and chemical shift data [115] were consistent with a model in which the zinc knuckles transiently interact with each other like “beads on a string” [82,115]; Figure 2B. However, the zinc knuckles of HIV-2 and the simian immunodeficiency virus (SIV) NC proteins appear to interact tightly with each other, in the absence of nucleic acids [103,104], and tight inter-knuckle packing was observed for HIV-1 NC when bound to RNAs with high affinity binding sites (see sections below). Residual dipolar couplings (RDCs) of an isolated NC domain interacting with DNA indicate that the two zinc knuckles of NC tightly bind with DNA, and tumble as a singular unit [109,110,114,116]. The NC protein from the mouse mammary tumor virus (MMTV) also contains two CCHC zinc knuckle domains, and NMR studies indicate that these domains do not interact with each other when free in solution [98]. Interestingly, while the proximal zinc knuckle of MMTV NC adopts the same folding observed for all other known retroviral zinc knuckles, the distal knuckle contains an additional C-terminal β-hairpin [98]. A similar reverse turn-like structure was observed for the distal zinc knuckle in the NMR structure of the Mason-Pfizer monkey virus NC protein [106].

### 2.2. MA Domain of Gag

NMR is a powerful tool in structural characterization of Gag’s MA domain [117,118,119,120,121], elucidating its roles in cytosolic trafficking [121,122,123,124,125], and unveiling MA–RNA interactions [126]. Assembly of HIV particles is facilitated by targeting the viral genome and ~12 copies of Gag to the assembly sites on the inner leaflet of the plasma membrane [29,30,31,127,128]. Roughly 2000 Gag molecules are subsequently anchored to the plasma membrane through Gag’s N-terminally myristoylated MA domain [129]. Given the challenges to prepare an in vitro myristoylation system, the initial structural characterization focused on unmyristoylated MA [myr(-)MA]. In 1994, Matthews et al. used multidimensional solution NMR to report the first structure of an HIV-1 myr(-)MA construct. This structure featured four ⍺-helices, as well as an irregular *β*-sheet arranged such that several basic residues were available on the protein surface to interact with plasma membrane assembly sites [117]. Structural similarity to the immune modulator interferon-*γ* led to the suggestion that the MA protein might operate as a dimer [117]. However, the NMR-derived solution structure of a similar construct presented by Massiah et al. in the same year countered this proposal, based on steric hindrance at the dimer interface due to an additional helix at the N-terminus [130]. Matthews et al. also observed this helix following further refinement [131]. Aside from this difference, the NMR-derived structures showed a similar globular fold, comprising five ⍺-helices, a short 3_10_ helical stretch, and a three-strand mixed *β*-sheet [130]. NMR studies of the 283 N-terminal residues of the Gag-precursor, which include full-length myr(-)MA [118], also revealed a similar fold. X-ray crystallography performed by the Sundquist group, again showed a similar globular fold, but unexpectedly indicated that MA trimerized in three different crystal lattices [132]. Comparison of the crystal and NMR structures revealed a roughly 6 Å displacement of the short 3_10_ helix participating in the trimer interface and ultimately led to the proposal that MA undergoes this structural rearrangement during assembly [133]. Recent virology work suggests that MA trimerization is necessary for recognition of the envelope glycoprotein for incorporation to new viruses [134,135,136]; therefore, this might also be the functional cause for multimerization.

The MA–plasma membrane interaction is dependent not only on the region of basic residues located on MA’s surface but also on N-terminal myristoylation [121,122,137,138], and efforts to characterize the structure of the native, myristoylated MA were realized by Tang et al., using solution NMR [119]. They found that MA adopts both myristoyl exposed and sequestered conformations, but unexpectedly showed that sequestration of the myristoyl group within the protein requires only minor conformational changes and does not alter its tertiary fold. Concentration-dependent chemical shift perturbations, combined with sedimentation equilibrium data, demonstrated a monomer–trimer equilibrium and proposed a model for MA trimerization via intermolecular myr–myr interactions [119] in a manner consistent with the earlier crystal structures [132]. Several subsequent NMR studies demonstrated that myristoylated MA proteins of other retroviruses, including HIV-2 [121] and feline immunodeficiency virus (FIV) [139,140], adopt similar structures to HIV-1 MA. Furthermore, NMR is used to demonstrate that myristoyl exposure is modulated by pH [141], with stabilization of a salt bridge between the protonated side chain of H89 and the acidic side chain of E12 promoting exposure.

**Figure 3:**
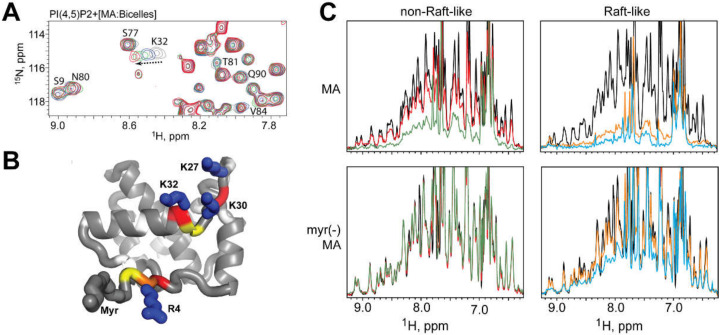
NMR studies of HIV-1 MA structure and membrane targeting. (**A**) ^1^H-^15^N HSQC spectra of ^15^N-labeled MA titrated into bicelles containing increasing mol% native PI(4,5)P_2_, reveal the binding mode of MA to plasma membrane mimetics. (**B**) Solution structure of MA determined by NMR. Residues showing large chemical shift perturbations in (**A**) are highlighted. (**C**) ^1^H NMR spectra of MA and myr(-) MA upon addition of liposomes with varying phospholipid compositions. Signal loss indicates binding. MA can bind non-Raft-like membranes due to myristoyl group interactions. Binding to Raft-like membranes is significantly enhanced. Adapted from [122] with permission.

NMR studies also played a key role in characterizing the targeting of MA to the plasma membrane during assembly. MA specifically targets phosphatidylinositol-(4,5)-bisphosphate (PI(4,5)P_2_) [123,142,143] in lipid rafts, on the inner leaflet of the plasma membrane [144,145,146]. Saad et al. first reported the NMR structure of HIV-1 MA bound to water-soluble truncated PI(4,5)P_2_ lipids [123], in which the 2′-fatty acid chain of di-C_4_-PI(4,5)P_2_ is buried in MA’s hydrophobic cleft in an “extended lipid” conformation, promoting myristoyl exposure [123]. A variety of other phospholipids were proposed to promote membrane binding in a similar manner, based on NMR studies, with lipid analogs containing truncated acyl chains [147]. Development of new NMR techniques employing mimetic membranes allowed re-examination in the context of full-length lipids; Figure 3 [122]. NMR titration experiments employing liposomes and bicelles designed to mimic plasma and viral membranes revealed that, while PI(4,5)P_2_ promotes MA–liposome interactions, no PI(4,5)P_2_-dependent chemical shift perturbation are seen in the hydrophobic cleft, calling into question the validity of the extended lipid binding model; Figure 3A. This work prompted consideration of a molecular-dynamics-derived membrane-binding model, in which several MA surfaces interact with PI(4,5)P_2_ head groups but the acyl chains of PI(4,5)P_2_ do not extend from the membrane; Figure 3B [122]. NMR was also used to characterize and explain the action of several MA point mutations that inhibit plasma membrane targeting by disruption of the tertiary structure [148], stabilization of the myr-sequestered conformation [120,122], or through removal of basic residues important for both PI(4,5)P_2_ discrimination and membrane binding, Figure 3C [122].

Recent studies demonstrated that MA interacts with cytosolic tRNAs [149], including the tRNA^Lys3^ isoform that is present in virions and serves as the primer for reverse transcription [149,150,151,152]. Chukkapalli et al. monitored the influence of MA–RNA interactions on assembly, using liposome flotation assays, which indicated that MA’s basic patch played a role in MA–RNA interactions and that this complex formation prevents MA from binding to PI(4,5)P_2_ [153]. Gaines et al. applied NMR and isothermal titration calorimetry (ITC) to probe the MA–tRNA interaction and determined that tRNA^Lys3^ interacts with residues in the basic patch region of MA. Interestingly, the affinity of MA for tRNA^Lys3^ was roughly one order of magnitude stronger than PI(4,5)P_2_-containing liposomes, and the NMR-based liposome binding assays revealed that tRNA^Lys3^ inhibited MA–PI(4,5)P_2_ interactions [126]. The binding affinity of MA to tRNALys3 is diminished by sample conditions that promote myristate exposure [123], which could also be promoted by Gag self-association [116]. Taken together, these studies point to a model suggesting that Gag–Gag interactions induced by NC binding to the RNA packaging signal might regulate premature binding to the plasma membrane and potentially support MA trimerization [126]. Thus, it is conceivable that genome recognition and Gag–Gag interactions might influence MA’s role in recruiting viral envelope glycoproteins to virions, further shaping the mechanism of HIV assembly [131].

### 2.3. Multi-Domain Gag Fragments

**Figure 4:**
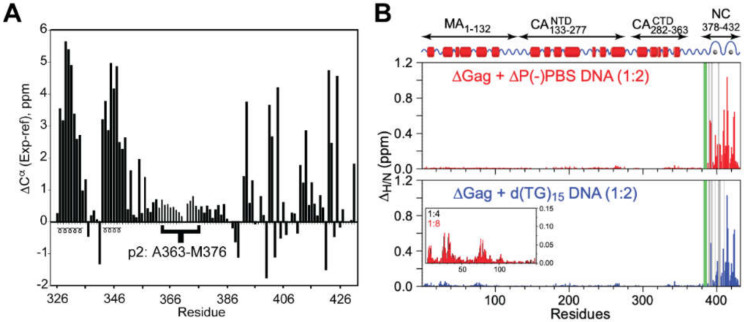
NMR studies of larger Gag constructs. (**A**) Plot of ^13^Cα chemical shift indices for SP1 (previously called p2, in braces) and adjacent residues in CA^CTD^-SP1-NC. Consecutive positive values are indicative of α-helical structure. (**B**) ^1^H/^15^N chemical shift perturbation profiles in the presence of DNA [Red—∆P(-)PBS, Blue—d(TG)_15_]. In the presence of ∆P(-)PBS, chemical shift perturbations are only seen within the NC domain. In the presence of d(TG)_15_, significant chemical shift perturbations are present within NC but some perturbations are present in regions of MA at higher DNA concentrations (inset). Adapted from [18] (Panel **A**) and [116] (Panel **B**), with permission.

The CA domain of Gag promotes assembly via formation of intermolecular Gag–Gag interactions. The C-terminal domain of CA (CA^CTD^) has a relatively weak propensity to form dimers in solution (dimerization K_d_ ~10 µM) [154], and both the N- and C-terminal domains participate in intermolecular contacts in assembled CA multimers [32]. To determine the influence of CA multimerization on downstream structure, NMR studies were conducted with a construct comprising CA^CTD^ through NC (CA^CTD^-SP1-NC) [18]. These studies revealed that SP1 is conformationally labile and exists as a mixture of unstructured (predominant) and helical (minor) states; Figure 4A. The structure of NC was unaffected by the presence of the upstream CA^CTD^ and SP1 domains. An even larger HIV-1 Gag construct (∆Gag) comprising domains spanning unmyristoylated MA through NC (myr(-)MA-CA-SP1-NC; ~100 kDa) was studied by Clore et al. using protein perdeuteration and transverse relaxation optimized spectroscopy (TROSY) NMR [155], to optimize sensitivity and resolution [116]. ^1^H-^15^N chemical shift perturbation mapping was used to explore the structural changes that occur as ∆Gag interacts with two different DNA oligonucleotides—∆P(-)PBS and d(TG)_15_ [116]. Upon addition of either oligonucleotide, some cross-peaks from the MA and NC domains exhibit chemical shift perturbations. Specifically, significant chemical shift perturbations occur within the NC domain as ∆Gag forms a 1:1 complex with the DNA oligonucleotide; Figure 4B. Minor ^1^H-^15^N chemical shift perturbations are observed within the MA domain, following saturation of the NC binding site, suggesting weak secondary interactions between the MA and the DNA oligonucleotides [116]; Figure 4B.

## 3. RNA Components Important for Genome Packaging

### 3.1. HIV-1

**Figure 5:**
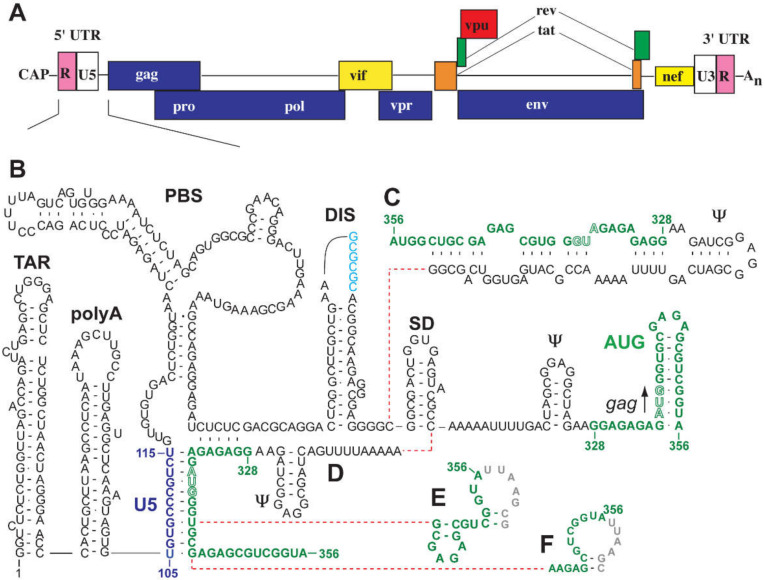
(**A**) Schematic of a representative HIV-1 transcript showing locations of the 5′-UTR and coding regions. (**B**–**F**) Five of the more than 20 different secondary structures predicted for the HIV-1 5′-leader, on the basis of nucleotide reactivity probing, phylogenetic analysis, and biochemical studies (highlighting variations in AUG residues, colored green). Adapted from [12], with permission.

Considerable effort was made over the past 30 years to understand the structure of the HIV-1 5′-leader and its role in genome packaging (reviewed in [12]). A combination of nucleotide accessibility mapping and biophysical studies of recombinant viral genomic RNAs suggest that discrete hairpin structures within the genomic RNA perform specific functions of the genomic RNA, including transcriptional activation, primer binding, dimerization, 5′ splicing activities, and genome packaging [156,157,158,159,160,161,162,163,164,165]. These hairpin structures are, from 5′ to 3′, the trans-activation region (TAR), 5′ polyadenylation signal (polyA), U5 region, primer binding site (PBS), dimer initiation site (DIS; sometimes called SL1), major splice donor (SD; or SL2), Ψ packaging signal (or SL3), and the AUG hairpin (or SL4) [71]; Figure 5. TAR plays an essential role in Tat-mediated transcription activation [166]. The 5′ polyA hairpin contains the polyadenylation signal, AAUAAA, but its function is not as well characterized as the 3′ polyadenylation signal, which recruits cellular machinery to catalyze the addition of a 3′ polyA tail. Some studies suggest that the structure of the 5′ polyA hairpin is involved in translation [167]. The PBS region contains the tRNA^Lys3^ primer binding site for initiation of reverse transcription [168,169,170] and might be involved in packaging cellular proteins (see below). The DIS contains a palindromic sequence that allows it to base pair with the DIS of another viral RNA molecule, initiating dimerization (see next section) [71]. The SD hairpin is the major splice donor site for all spliced variants [171], thus all spliced constructs contain a common exon upstream of this site [172]. The Ψ hairpin contains the high affinity binding site for the viral NC protein, and the AUG hairpin (SL4) contains the start codon for Gag and Gag-Pol translation [71].

Historically, there is little agreement regarding the structures of residues within the 5′-leader that play a role in genome packaging. In fact, more than 20 different structures were proposed for the HIV-1 5′-leader, based on combinations of nucleotide accessibility mapping and phylogenetic analyses [157,158,159,162,173,174,175,176,177,178,179,180]. For example, residues that overlap the *gag* start codon (which are important for both genome packaging [181] and RNA dimer stability [182]) were proposed to form a hairpin, to base-pair with residues of the U5 element [161,162], or to adopt other conformations [12]; Figure 5. In vivo nucleotide reactivity mapping supported multiple structures for AUG and other elements that are essential for packaging, without consensus [12,163,164,165]. One important advantage of NMR, relative to chemical probing methods, is that it enables direct detection and identification of different structures that might exist within an equilibrium mixture of species. Applications of NMR to different domains of the HIV-1 5′-leader and to the intact leader are described below.

#### 3.1.1. DIS

**Figure 6:**
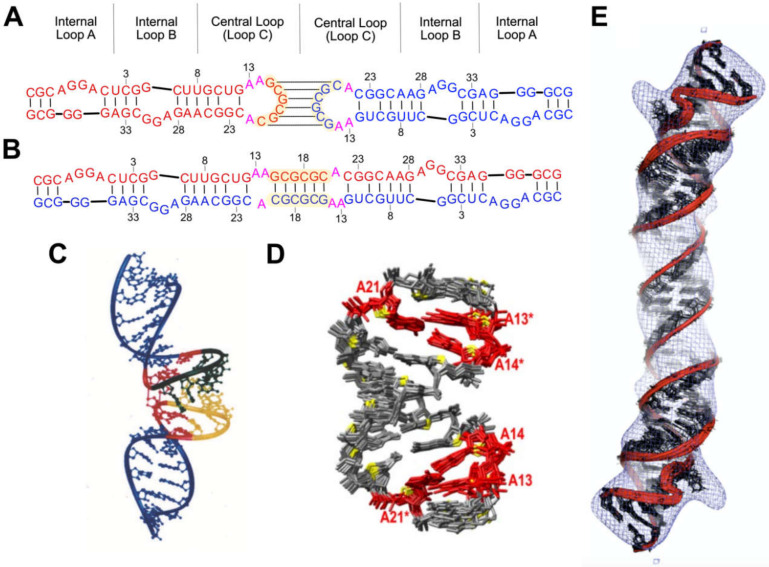
Kissing and extended duplex forms of dimeric DIS. (**A**,**B**) Secondary structures of the HIV-1_NL4-3_ DIS element in kissing (**A**) and extended duplex (**B**) conformations. Individual RNAs denoted in red and blue. The palindrome and flanking purines of the apical loop (Loop C) are colored yellow and pink, respectively. Residue numbers correspond to that of a truncated construct utilized in (**D**). (**C**) Solution NMR structure of a DIS kissing dimer. (**D**) Solution NMR structure of the extended dimer. Flanking purines (red) stack to form a zipper motif. (**E**) Structure of the extended duplex form of DIS, as determined by a hybrid NMR/cryo-EM approach. Panels (**C**–**E**) reproduced from [202,203,204], respectively, with permission.

Packaging of the HIV-1 genome is intrinsically dependent on RNA dimerization [6,9,12,54,183,184,185,186,187]. Studies showed that residues of the DIS pseudopalindrome (Figure 5B–F) are required for dimerization of RNAs prepared by in vitro transcription [180,188,189,190,191] or purified from virions [192]. The DIS residues can adopt a hairpin structure with a central loop that contains a six-nucleotide palindrome and is capable of forming an intermolecular “kissing” dimer interface [193,194,195]; Figure 6A. As the DIS is a pseudopalindrome, it is also capable of forming an extended duplex structure (Figure 6B) possibly via formation of an initial kissing species [192,196,197,198,199]. The stem of the hairpin also contains two conservatively substituted bulges (internal loops A and B, Figure 6A,B) [200,201].

The loop–loop kissing interactions were structurally characterized by both NMR [202,205,206,207,208,209] and X-ray crystallography [210]. All structures exhibited coaxial intermolecular stacking of the helices, with variations in the degree of symmetry between the stacked hairpins [202,205,210]. These discrepancies were attributed to variations in sample conditions and structural restraints, but crystal lattice interactions that favor linear helical structures might also be a factor. HNN-COSY experiments confirmed the presence of intermolecular Watson–Crick G-C base pairs [205], and aromatic and imino proton NOEs were consistent with an underwound A-helical structure at the kissing interface [202,205,206,207]. The NMR data also indicated that the purine residues flanking the palindrome were stacked with residues of the loop residues; Figure 6C [202]. These findings contradict earlier crystal structures in which flanking purines are not stacked within the helix but instead adopt a bulged out conformation [210]. Additional NMR studies revealed that these residues might adopt a dynamic equilibrium between internally stacked and bulged out (minor population) conformations [205,206]. As discussed below, this conformational equilibrium might play a role in the conversion between kissing and extended duplex conformations [208].

Oligonucleotides that adopt the kissing dimer structure can be converted to a more thermodynamically stable extended dimer [180,189,196,211,212] by incubation at 55 °C [203,211,213], or by incubation at lower temperatures in the presence of NC [69,190,214]. Extended duplex DIS oligonucleotides can be prepared by boiling and slow cooling RNA samples, the inclusion of salts, or by use of longer hairpin constructs [204,207]. Three dimensional structures were determined by X-ray crystallography [215], NMR [203,204,207,213,216,217], Figure 6D, and a hybrid NMR/cryo-EM approach [204]; Figure 6E. The extended conformation largely mimics the dimer structure described above, but differs from the kissing species in the region flanking the palindrome, where purines instead exhibit a zipper-like motif with additional stacking contacts that stabilize the extended duplex; Figure 6D [203,207].

Residues of the internal loops (bulges) adopt similar structures in the kissing and extended duplex forms of the DIS dimers [207]. NMR studies defined the non-canonical nature of both internal loops for a number of native and mutant DIS constructs [201,209,217]. Internal loop A adopts a well-defined S-turn structure [201,204], similar to a turn observed in the HIV-1 rev response element (RRE) [218,219]. Internal loop B signals were broadened in some studies, consistent with conformational dynamics and disorder [201,217]. However, the signals were well-resolved in a subsequent NMR study that revealed internally stacked bases, possibly due to intrinsic differences in the constructs studied [207]. These internal loops are thought to serve as potential sites for NC binding [201,217] and helix unwinding, as the RNA converts from the kissing to the extended duplex (see below) [220].

NMR methods are used to monitor the mechanism of DIS conversion from the kissing to the extended duplex structure, as well as the temporal formation and discrimination of intermolecular contacts [212,221,222]. Differential behavior of imino protons identified nucleotide-specific unwinding events upon temperature- and NC-induced structural conversion [206,223]. ^1^H-^15^N heteronuclear single quantum coherence (HSQC) studies of wild type and mutant NC constructs identified the N-terminal residues and residues flanking the zinc-knuckle domains as critical to this conversion [224,225]. RDCs, dynamically decoupled spin relaxation, and chemical shift mapping NMR studies enabled direct detection of internal motions within the DIS [220]. These motions were found to be differentially greater for residues “above” internal loop B (closer to the palindrome), and arrested by Mg^2+^, suggesting a mechanism through which Mg^2+^ regulates the kissing loop unfolding, to prevent premature conversion to the extended duplex structure, in the absence of NC. Further work also established this mechanism of interconversion to be pH-dependent, upon protonation of a flanking purine residue [208].

Takahashi et al. utilized mixed samples containing unlabeled and ^15^N-labeled RNAs, to distinguish between kissing and extended duplex dimer interfaces in DIS fragments [212]. ^15^N splitting patterns observed for Watson–Crick imino protons confirmed that “weak dimers” contain a kissing interface, whereas the dimers that are more thermally stable contain an extended duplex intermolecular interface.

#### 3.1.2. Ψ-hairpin

Early mutagenesis studies that deleted residues spanning from the major splice donor site to the *gag* initiation codon, demonstrated that these residues are essential for wild-type packaging efficiency [226,227,228,229]. RNAs containing this 46-nt region are capable of promoting the packaging of heterologous RNAs into virus like particles [173]. A conserved stretch of residues within this region were predicted to form a conserved hairpin that is historically called the Ψ-hairpin (or SL3) [156,157,159]; Figure 5B–F. The role of Ψ hairpin sequence and structure in genome packaging was probed by mutagenesis. Destabilization of the stem of the Ψ-hairpin significantly reduced dimerization and encapsidation efficiencies, but wild-type efficiencies were rescued by the revertant mutations [175,230,231,232,233]. This finding suggested the structure of the Ψ hairpin, rather than its sequence or downstream GA-rich residues, is critical for genome dimerization and packaging [233]. Other in vitro binding studies with larger Ψ-containing RNA fragments and Gag constructs, support a role for Ψ-NC interactions in Gag binding and packaging [234,235]. Although NC is capable of binding tightly to the conserved GGAG tetraloop of Ψ [109] (see below), substitution of the GGAG loop by GCUA or AAGA did not significantly affect packaging or replication [233]. Recently, NMR and isothermal titration calorimetry (ITC) studies revealed that the weakly base-paired [UUUU]:[GGAG] helical region in the lower stem of the Ψ hairpin, serves as the initial high-affinity NC binding site (K_d_ ~40 nM) and that structural rearrangements induced by NC binding are required for in vivo RNA packaging [236]. Although considerable effort focused on the structure and NC interactions of the Ψ-hairpin [237,238], it is now clear that competitive RNA packaging requires a much larger portion of the leader (see below) [40].

#### 3.1.3. AUG and U5:AUG

Mutagenesis and other studies showed that the nucleotides surrounding the *gag* initiation codon are crucial for genome dimerization, Gag binding, and packaging [157,158,175,231,239,240,241]. These residues (AUG) were originally predicted to form a hairpin (Figure 5B) and NMR studies confirmed that oligonucleotides with sequences of AUG form hairpins with structurally well-defined GNRA-type tetraloops (G = guanosine, N = any nucleotide, R = purine, A = adenosine) [242,243]. Phylogenetic studies led to proposals that this stretch of residues instead forms base pairs with residues of an upstream element commonly called U5, thus, forming a U5:AUG helix, Figure 5D [161]. Subsequent structural probing and phylogenetic analyses were also consistent with U5:AUG pairing [162]. Although Paillart et al. did not find evidence for U5:AUG interactions or global RNA rearrangement in cells or in viral particles, using chemical probing [163], Weeks et al. suggested that U5:AUG base pairing does exist in the viral genomic RNA in virions, in transfected cells, and in vitro [164] (although other U5:AUG structures were subsequently proposed by this group [165]).

Long-range U5:AUG base pairing was later supported by NMR studies. 2D NMR analysis showed that an 11-mer U5 RNA can disrupt the AUG stem-loop structure, to form intermolecular U5:AUG duplex [114]. More recently, the U5:AUG interaction was confirmed in the intact leader (712 nt) by NMR using a segmental labeling approach [71]. The unlabeled 5′-leader fragment (residues 1-327) and the ^13^C-labeled 3′-fragment (residues 328–356) were enzymatically ligated to facilitate the direct probing of the AUG conformations in the context of the large, intact, leader RNA. When the RNA sample was incubated in physiological-like buffers that promote dimerization, NMR spectral changes consistent with an AUG structural conversion from a hairpin to a U5:AUG duplex were observed. The U5:AUG base pairing was then probed directly, using an NMR method for probing long-range interactions through adenosine–interaction detection (lr-AID; see below) [71]. An upfield adenosine signal corresponding to the central adenosine in the UAA triplet of the lr-AID element was observed, providing direct evidence for the presence of U5:AUG base pairing [71]. The three dimensional structure of U5:AUG in a larger fragment of the HIV-1 leader was subsequently reported (see below) [41].

#### 3.1.4. TAR

The TAR element is a 59-nt sequence located at the 5′-end of all HIV-1 nascent viral transcripts and plays an essential role in Tat-mediated transcriptional activation [244,245,246,247,248]. Mutagenesis studies led to proposals that TAR is also involved in a variety of functions, including dimerization [249,250], strand transfer during reverse transcription [251], translation [75], packaging [239,252,253,254], HIV-1 derived microRNA (miRNA) during latency [255], and even the growth and progression of some cancers [256]. Some studies suggested the stability of the lower stem of TAR is important for RNA packaging [252,253]. In these studies, packaging efficiency was reduced by mutations designed to destabilize base pairing in the lower stem of TAR, and was recovered by compensatory mutations that stabilized the lower stem [254]. Das et al. found that viral replication can occur efficiently upon complete deletion of TAR when TAR is not needed for transcription, and that its sequence can be replaced by any sequence that can form a stable stem-loop at the 5′ of the transcript, suggesting the structure rather than sequence of TAR is important for packaging [257]. However, viral packaging studies can be complicated by a variety of factors, such as dominant negative effects [257], lack of viral accessory proteins [258], non-selective packaging [37,38,259,260], high RNA concentration [40], and improper mutation design that leads to misfolding [12,71]. In 2012, Heng et al. showed that the deletion of TAR did not significantly impair leader dimerization or vector RNA packaging, at least in a non-competitive packaging assay [40].

HIV-1 TAR adopts a hairpin structure that consists of two A-form helical domains connected by a three-pyrimidine residue bulge that binds to the Tat protein [261,262,263,264] and an apical 6-nt loop that binds to the cyclin T1 subunit of the cellular positive transcriptional elongation factor (pTEFb) during transcription [265,266]; Figure 5B–F. The first TAR and TAR-Tat complex structures determined by NMR revealed the structural elements that are critical for Tat binding—the most 5′ residue in the bulge (U23), the two base-pairs immediately downstream from the bulge (G26-C39 and A27-U38), and three phosphate groups [267,268]. In the free TAR hairpin, the helix axis is bent by an internal stacked bulge structure [267]. This result was supported by subsequent heteronuclear multi-dimensional NMR studies [269]. TAR undergoes a conformational change when binding to an arginine analog that mimics the TAR recognition site in the Tat protein, where the three bulge nucleotides become unstacked, and the two helix stems are coaxially stacked [267]. In addition, a base-triple forms between U23 and A27-U38, to stabilize the interaction of arginine with G26 and phosphates in the major grooves [267,268]. The base-triple model was supported by later NMR work [270,271,272,273]. NMR-based structural studies revealed that the apical 6-nt loop forms a deep binding pocket with bulge residues, when induced by high affinity Tat binding [271,273]. Conformational shifts in free and bound TAR states show that TAR undergoes extensive dynamic rearrangements related to its functions. More recently, D’Souza et al. used NMR to identify interactions between a large fragment of Tat (the Tat RNA binding domain, RBD) and both TAR and 7SK, a cellular small nuclear RNA that regulates transcriptional elongation [274]. These studies suggest that Tat and TAR evolved dual structural mimicry of cellular HEXIM and 7SK, respectively, providing a mechanism for promoting elongation of stalled HIV-1 transcripts [274]. TAR dynamics were extensively studied for HIV inhibitor development [272,275,276,277].

#### 3.1.5. PBS

The function of the 5′ PBS is well characterized as the annealing site for tRNA^Lys3^—the packaged host transfer RNA (see section on matrix) that serves as the primer for viral reverse transcriptase [152]. NC mediates the annealing between the 3′ end of tRNA^Lys3^ and a complementary 18-nt PBS fragment (see section on NC–RNA interactions) [278]. An additional eight-nucleotide binding site located upstream of the PBS in the U5 region, called the primer activation site (PAS), anneals with the TΨC arm of the transfer RNA, before reverse transcription is initiated [279]. NMR studies revealed the formation of an intramolecular helix, upstream of the PBS, and significant remodeling of the transfer RNA, to accommodate the base pairing between the PAS and TΨC arm [168,280]. The formation of this structural element was shown to be a kinetic block to reverse transcription [281].

The PBS might also play a role in packaging cellular RNA helicase A (RHA) into virus particles [282,283,284,285]. RHA-knockouts significantly lower reverse transcription efficiencies in viral particles [282,283,284,285], and it appears that RHA might function in coordination with NC to promote reverse transcription elongation, by promoting primer extension through the intramolecular helical structure formed upon tRNA^Lys3^ annealing. The recognition mechanism of RHA by the 5′-leader of HIV-1_NL4-3_ during viral assembly was recently characterized via a combination of NMR and ITC studies, and it was found that RHA and NC noncompetitively interact with the leader RNA [286]. Chemical shift perturbation of A220 in the 5′-leader in NMR titration experiments showed that RHA specifically binds to the PBS region. Additionally, these findings were corroborated through in vivo mutagenesis studies showing that A220-to-cytidine substitution significantly reduced RHA packaging and viral infectivity [286].

#### 3.1.6. Core Encapsidation Signal of HIV-1

**Figure 7:**
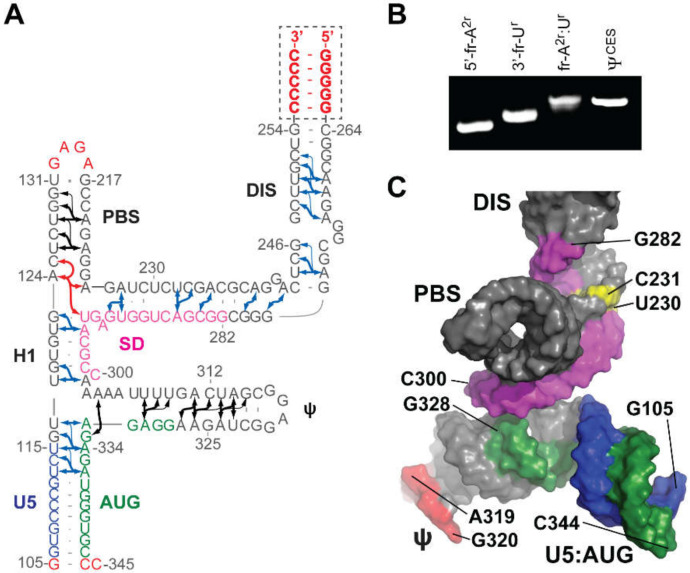
NMR structure of HIV-1_NL4-3_ Ψ^CESm^. (**A**) Fragment-annealed sample used to identify long-range adenosine-H2 detected NOEs (denoted by arrows). Non-native residues are shown in red; U5, blue; AUG, green; and SD, pink. (**B**) Native polyacrylamide gel electrophoresis showing non-covalent annealing of differentially labeled 5′- and 3′- fragments. (**C**) Representation of the tandem three-way junction NMR structure adopted by Ψ^CESm^ (colors match panel **A**; conformationally dynamic nucleotides, yellow; Ψ tetraloop, orange). Adapted from [41], with permission.

NMR was used to help identify a minimal region of the 5′-leader capable of directing RNA packaging. Deletions and mutations that did not significantly affect NC binding or RNA misfolding in vitro were employed in RNA packaging experiments. These studies revealed that the TAR, polyA, and PBS elements are not required for packaging, and identified a minimal ~260 nt region of the 5′-leader that is capable of efficiently promoting the packaging of heterologous vector RNAs (Core Encapsidation Signal, Ψ^CES^) [40] (see below). Structural studies of Ψ^CES^ were facilitated by substituting the dimer-promoting GC-rich loop of the DIS hairpin by a GAGA tetraloop (Figure 7A), effectively cutting the symmetric dimer in half to improve spectral sensitivity and resolution associated with more rapid molecular tumbling [41]. Even with this modification, the size of the resulting RNA construct (155 nt, Ψ^CESm^) was five times larger than the average size of RNA structures previously solved by NMR. Additionally, nucleotide-specific deuterium labeling did not provide sufficient resolution to enable complete assignment of the Ψ^CESm^ spectra. Resolution was further improved using a novel approach involving the non-covalent annealing of differentially ^2^H-labeled RNA fragments [41]; Figure 7B. Assignment and validation of NMR signals were facilitated by comparisons of chemical shifts with average database values, using software that correlates signals based on expected NOE patterns [287,288,289,290]. An improved understanding of the impact of NMR restraints and forcefields on the RNA structure, complemented these tools to help determine the 3D structure of the Ψ^CESm^ [41,291]; Figure 7C. Unexpectedly, the RNA adopted a tandem three-way junction structure in which residues of the SD did not form a hairpin but instead were sequestered by long-range base pairing. Several guanosines that were either held in exposed or weakly base-paired conformations were shown by mutagenesis to be essential for high affinity NC binding and RNA packaging. The Ψ^CESm^ structure also explains how HIV-1 selectively packages its unspliced genome [55]. Residues immediately downstream of the major splice site are integral to the formation of the tandem three-way junction structure, and differences in spliced mRNA sequences derived from 3′ exons would preclude formation of the packaging, competent, three-way junction structure.

#### 3.1.7. NMR Studies of the Intact HIV-1 Leader

**Figure 8:**
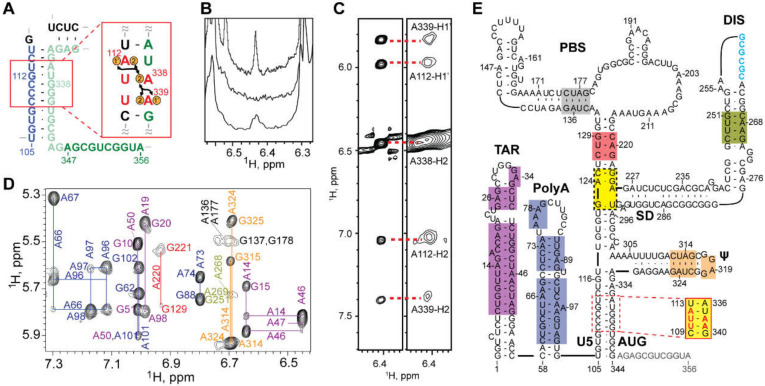
NMR studies of the intact, dimeric HIV-1_NL4-3_ 5′-leader. (**A**) Illustration depicting mutations in the lr-AID substitution and ^1^H-^1^H NOEs. (**B**) Region of 1D ^1^H NMR spectra showing (top) the native TAR A46-H2 signal, (middle) A46G substitution, (bottom) A338-H2 signal observed for the lr-AID substitution. (**C**) 2D ^1^H-^1^H NOESY spectra of the same A338-H2 signal observed an isolated U5:AUG hairpin (left) and intact dimer (right) containing the lr-AID substitution. (**D**) Region of the 2D ^1^H-^1^H NOESY spectrum of A^2r^G^r^C^r^-labeled dimeric HIV-1_NL4-3_ RNA; color-coding matches the elements shown in (**E**). (**E**) Secondary structure of the HIV-1_NL4-3_ in its DIS-exposed, dimer-promoting state. Color-shaded boxes denoted resolved and assigned 2D ^1^H-^1^H NOESY signals; yellow denotes sites that were only assignable in mutant constructs using truncated constructs or lrAID substitution. Panels (**A**–**C**) adapted from [71] and panels (**D**,**E**) from [221], with permission.

The 5′-leader of the NL4-3 strain of HIV-1 (residues 1-356; Figure 8A) forms a dimer with molecular weight of ~230 kDa, and it was surprising that a system of this size would give rise to interpretable NMR spectra [71]. The unexpectedly narrow NMR signals indicated that the RNA adopts a structure with independently folded subdomains that move with rotational correlation that is normally associated with much smaller molecules. Spectra obtained for nucleotide-specific ^2^H-labeled samples enabled direct detection of several of the predicted secondary structural elements located within the intact, dimeric 5′-leader; Figure 8A,B [40,71,221]. ^1^H-^13^C NMR studies were conducted with a partially ^13^C labeled leader RNA prepared by enzymatic ligation of an unlabeled 5′ -fragment and a ^13^C-labeled 3′-fragment [71]. Under low salt conditions where a portion of the RNA exists as a monomer, NMR signals were readily detected for the labeled residues. The ^1^H-^13^C NMR correlation spectra matched those of an isolated AUG hairpin, indicating that the AUG element adopts a hairpin structure in the monomeric leader. Under physiological-like buffer conditions that favor the dimer, several ^1^H-^13^C NMR signals disappeared due to extensive line broadening, and the remaining signals exhibited linewidths and chemical shifts consistent with an unstructured conformation. These findings confirmed that the AUG residues adopt an alternate (but largely undefined) conformation in the dimer.

The ^2^H-edited NMR studies led to the discovery that the helical structure adopted by a rare triplet of base pairs, [A^i^A^i+1^U^i+2^]:[A^j^U^j+1^U^j+2^], gives rise to well resolved A^i+1^-H2 NMR signals. With this knowledge, leader RNAs were engineered to include a [AAU]:[AUU] element in the regions predicted to adopt A-form helical elements. This approach (lr-AID) enabled the direct detection of U5:AUG base pairing in the 230 kDa dimeric HIV-1_NL4-3_ leader [71]; Figure 8C–E. The approach also revealed that the loop nucleotides of the DIS element form base pairs with an upstream U5 region in the monomeric form of the RNA. This base pairing was also confirmed in NMR studies of the native HIV-1_MAL_ leader (see below) [75]. ^2^H-edited NOESY NMR spectra were also obtained for HIV-1_NL4-3_ leader RNAs containing deletions or loop substitutions and compared with spectra of the intact, dimeric leader [40]. Mutations that did not cause global structural remodeling (determined via NMR) were employed for RNA packaging experiments. These studies revealed that the TAR, 5′ polyA, and PBS domains are not required for efficient packaging. Efficient packaging in a non-competitive packaging assay was achieved by a 155-nucleotide region of the leader comprising the U5, DIS, SD, the Ψ-hairpin, and AUG elements [40] (Ψ^CES^, see above).

**Figure 9:**
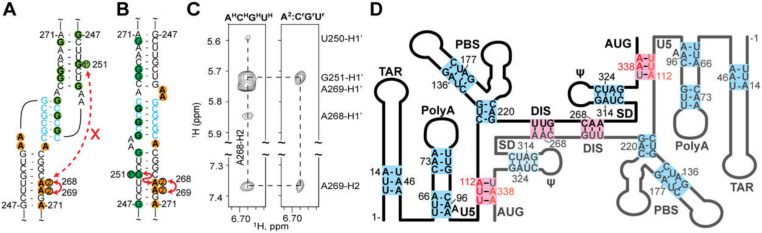
Discrimination between kissing and extended duplex base pairing in the HIV-1_NL4-3_ 5′-leader by ^2^H-edited lr-AID NMR. (**A**,**B**) Cross-strand NOEs between A268-H2 and G251-H1′ establish the nature of the intermolecular interface in the kissing (**A**) versus extended duplex (**B**) interfaces (green dots = G^r^ labeling; orange dots = A^2^ labeling). (**C**) A268-H2 to G251-H1′ NOEs in both A^2^G^r^ and A^2^:G^r^C^r^U^r^ support an extended duplex interface. (**D**) Summary of intermolecular (pink) (U5:AUG and DIS) and intramolecular (blue) (TAR, PolyA, PBS, Ψ) base pairings detected by NMR. Reproduced from [221], with permission.

^2^H-edited NMR was additionally used to probe the nature of the dimer interface in the intact HIV-1_NL4-3_ leader [221]. Separately prepared HIV-1_NL4-3_ leader RNAs containing A^2^-labeling (sample 1) (superscripts denote sites of protonation, all other sites deuterated; e.g., A^2^ = adenosines protonated only at the C2 position, G^r^ = guanosines protonated only at ribose carbons) and a G^r^-labeling scheme (sample 2) were mixed, and 2D NOE spectroscopy (NOESY) data were obtained. A268-H2-to-G251-H1′ NOE signals were detected for a sample containing an equimolar mixture of A^2^-leader and C^r^G^r^U^r^-leader RNAs, which is only possible if the leader adopts the extended duplex conformation; Figure 9A–C. Mixed samples containing other G^r^ labeling schemes showed similar results. In contrast, experiments probing long-range interactions in the TAR, polyA, PBS, and Ψ-hairpins gave results consistent with intramolecular base pairing in these regions of the leader; Figure 9. 1D NMR experiments revealed that the extended interface forms rapidly (within ~10 min) and does not require the presence of NC or other RNA chaperones. More recent single molecule FRET experiments with a 5′-leader fragment containing native U5 and AUG sequences are consistent with the U5: AUG base paired structure observed by NMR, and indicate that the RNA exists as an equilibrium mixture of structures [292].

**Figure 10:**
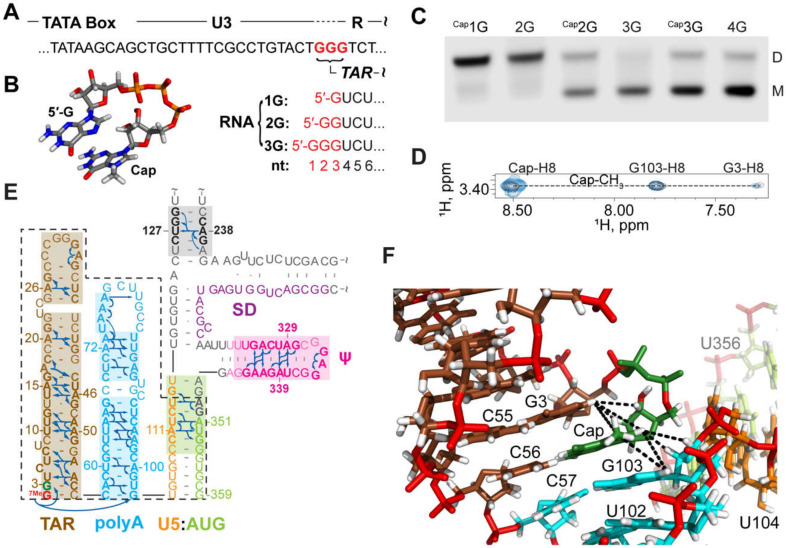
Heterogeneous transcriptional start site usage modulates HIV-1_MAL_ RNA dimerization and function. (**A**) Three guanines (red) can serve as alternative transcription start sites. (**B**) Transcripts are co-transcriptionally capped by 7-methylguanosine. (**C**) Effect of 5′-guanosines and capping on 5′-leader dimerization. (**D**) Portions of 2D NOE spectra showing similarities of Cap-CH_3_ to Cap-H8, G3, and G108 NOEs observed for G^8^ intact dimer and the Capped TAR-polyA-U5AUG (^Cap^1G-leader^TPUA^). (**E**) Assigned A-H2 NOEs and deduced secondary structure; discrete functional elements differentiated by color. Dashed lines denote residues of the ^Cap^1G-leader^TPUA^ construct used for high-resolution structural studies. (**F**) Structure of the ^Cap^1G-leader^TPUA^ showing end-to-end stacking of the TAR and polyA helices and sequestration of the Cap. Adapted from [75], with permission.

The above studies provided insights into the secondary structures adopted by selected regions within the HIV-1_NL4-3_ 5′-leader. Unfortunately, the high propensity of this leader to form dimers made it difficult to obtain more extensive NMR data for the monomeric form of the RNA, due to the high sample concentrations required for NMR studies (typically > 50 µM). In studies of in vitro transcribed 5′-leader RNAs from different strains of HIV and SIV, it was discovered that the dimerization propensity of the simian immunodeficiency virus (SIV_cpzUS_ strain) leader, which contains a GUGCAC palindrome in the dimer promoting DIS loop, is substantially lower than that of the HIV-1_NL4-3_ leader [293]. Therefore, studies shifted to the 5′-leader of HIV-1_MAL_, which also contains a GUGCAC palindrome and exhibits a dimerization propensity similar to that of SIV_cpzUS_ [75]. Thus, the HIV-1_MAL_ 5′-leader could be studied by NMR in its monomeric (low ionic strength) and dimeric (PI buffer) states [75]. In addition, in the course of studying 5′-leader RNAs from other lentiviruses [293], it was discovered that RNAs beginning with more than two guanosines at their 5′ ends exhibit stronger propensities to remain monomeric. Like all eukaryotic cellular mRNAs, HIV-1 transcripts are co-transcriptionally capped by a 5′-5′ triphosphate reverse-linked 7-methylguanosine moiety, shortly after initiation of the RNA polymerase II dependent transcription [294,295,296,297]; Figure 10. Interestingly, the cap moiety influences dimerization in a manner similar to that of an additional guanosine. Thus, non-capped 2G and capped-1G 5′-leader transcripts readily form dimers, whereas non-capped 3G/4G transcripts and capped 2G/3G transcripts, all preferentially form monomers [73].

A review of published retroviral DNA sequences [73] revealed that the transcripts were predicted to begin with one, two, or three 5′-guanosines (1G, 2G, and 3G, respectively), a consequence of inconsistencies in defining the position of the HIV-1 transcription start site (TSS); Figure 10 [72,296,298,299]. The TSS is genetically defined as the first residue in the Repeat (R) element of the Long Terminal Repeat (LTR), and retroviral proviruses typically contain a conserved stretch of three Gs at the U3/R junction. Early attempts to identify the actual TSS led to inconsistent conclusions [296,298,299], but more recent studies revealed that all three guanosines function as transcriptional start sites during viral replication [72,73]. Importantly, the 1G transcripts are specifically packaged into virions whereas the 2G/3G transcripts are retained in cells and are enriched on polysomes [73]. These findings supported a model in which 1G transcripts adopt a structure that promotes dimerization, NC binding, and packaging, whereas the 2G/3G transcripts adopt an alternative structure that inhibits dimerization, NC binding, and packaging [73].

To understand how transcriptional addition of as few as one or two 5′-guanosines influence the dimerization properties and function of ~9 kilobase HIV-1 transcripts, the secondary structures of 5′-capped HIV-1_MAL_ 1G, 2G, and 3G 5′-leader RNAs were probed by ^2^H-edited NMR [75]. The dimeric 5′-capped-1G leader RNA adopted a secondary structure consistent with that of the non-capped 2G HIV-1_NL4-3_ leader; Figure 10. It is important to note that signal diagnostic of the U5:AUG helix were detected in the native HIV-1_MAL_ transcript, unlike the HIV-1_NL4-3_ transcript that required mutagenesis (lr-AID; see above) for U5:AUG detection. It is also noteworthy that signals were not resolved for the H1 helix observed in the HIV-1_NL4-3_ Ψ^CES^ structure. It is possible these signals were obscured by spectral overlap, and future studies to test for the presence of a tandem three-way junction structure similar to that observed for the HIV-1_NL4-3_ Ψ^CES^ are warranted. The NMR spectra for the intact 5′-capped 1G HIV-1_MAL_ leader also exhibited evidence that the cap residue is sandwiched between the TAR and polyA helices. NMR studies of a leader fragment comprising the capped TAR, polyA, and U5:AUG helices confirmed that the TAR and polyA helices are stacked in an “end-to-end” manner; Figure 10 [75].

**Figure 11:**
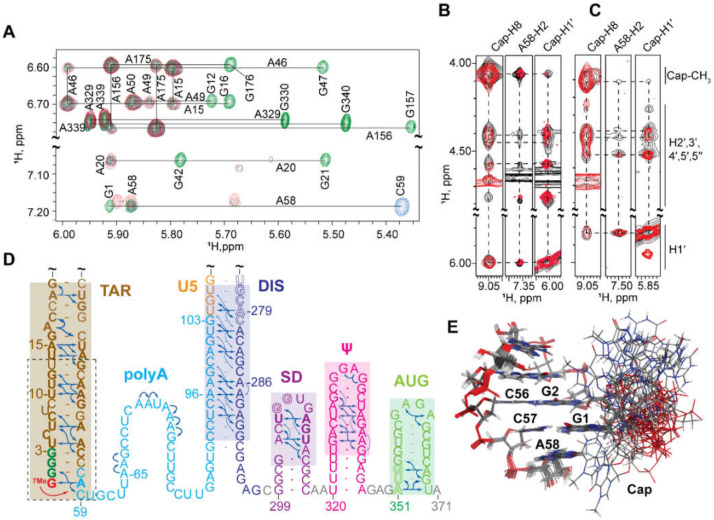
NMR and structural findings for the monomeric ^Cap^3G and ^Cap^2G forms of the HIV-1_MAL_ leader. (**A**) Regions of the 2D ^1^H-^1^H NOESY spectra for the non-capped 3G- leader^371^ (A^H^, black; A^2r^G^r^, green; A^2r^C^r^, blue; G1^H^A^2r^, red) used to make secondary structure assignments shown in D. (**B**) Overlapped 2D ^1^H-^1^H NOESY spectra for A^2^-^Cap^2G-leader^371^ (black) and TAR fragment ^Cap^2G-TAR^m^ (dashed outlined residues in panel D with U13 and G47 connected by a GAGA tetraloop) (red), showing that the cap methyl group is in close proximity to A58. (**C**) Similar results were obtained for ^Cap^3G-leader^371^ (black) and a ^Cap^3G-TAR^m^ RNA (red). (**D**) NOEs (arrows) and secondary structure of monomeric Cap-3G HIV-1_MAL_ leader. (**E**) Structure of a ^Cap^3G-TAR^m^ showing the unstructured Cap. Adapted from [75], with permission.

**Figure 12:**
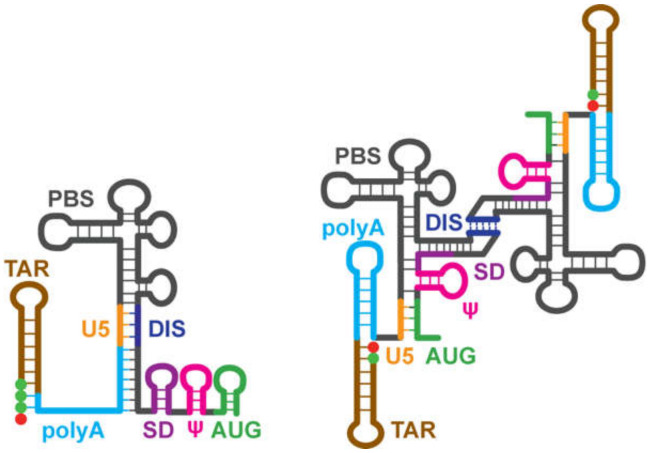
Influence of 5′-guanosine number on RNA structure. Capped RNAs containing two or three 5′-guanosines (**left**) adopt a monomeric structure that exposes the cap and enables RNA processing and metabolism, whereas those with a single capped G (**right**) adopt a cap-sequestered conformation that promotes dimerization and packaging (Cap = red sphere, guanosines = green spheres). Adapted from [75], with permission.

^2^H-edited NMR studies performed for the monomeric forms of the capped 2G/3G MAL leader RNAs, revealed extensive structural remodeling, compared to the capped 1G transcript [75]. Residues of the DIS palindrome were found to base pair with U5 in a manner similar to that observed for the NL4-3 leader (although the HIV-1_MAL_ data were obtained for the native RNA whereas the HIV-1_NL4-3_ studies were conducted with mutants using the lr-AID NMR method [71]). The remainder of the DIS residues did not form a hairpin but were instead found to from long-range base pairs with upstream residues of polyA, Figure 11. Importantly, the residues of polyA did not adopt a hairpin structure, as observed for the dimeric capped 1G RNA. Instead, a majority of the polyA residues exhibited only sequential NOEs (no long-range NOEs) and chemical shifts consistent with a disordered, non-base paired structure; Figure 11. Furthermore, unlike the dimeric capped 1G leader, in which the cap residue is sandwiched between the TAR and polyA helices, the cap residue of the capped 2G/3G leader RNAs appeared exposed and disordered, a finding confirmed by 3D structural studies of a capped TAR fragment RNA [75]. Biochemical studies revealed that cellular cap binding proteins readily bind the capped 2G/3G MAL leader RNAs, but do not bind to the capped 1G leader, suggesting that cap sequestration/exposure, modulated by heterogeneous TSS usage, might play a role in establishing the function and fate of the HIV-1 transcript [75]; Figure 12.

### 3.2. Other Retroviruses

#### 3.2.1. Moloney Murine Leukemia Virus (MoMuLV)

MoMuLV is an evolutionarily distant C-type retrovirus that was extensively studied as a model for retroviral genome encapsidation, and as a potential vector for therapeutic gene delivery [300]. A 350-nt “Ψ site” was initially identified by Mann et al., when deletion of the region between the *env* splice site and *gag* start codon abrogated the MoMuLV genome packaging [301]. Although the Ψ-site is required for packaging, upstream [302] and downstream [303] residues appear to be important for optimal packaging efficiency. The most efficient packaging was achieved with a region (Ψ^+^) containing the Ψ site and an additional 474 nt of the *gag* coding region [304]. We note here that some HIV-1_NL4-3_ vectors with non-native residues downstream of the leader were also poorly packaged. This was originally attributed to a role of coding residues in packaging; however, more recent studies indicate that the non-native residues might disrupt the structure of the leader and thereby inhibit packaging [42]. It is, thus, possible that the vectors employed in these early MoMuLV packaging experiments might also adversely affect the structure and function of the packaging signal.

**Figure 13:**
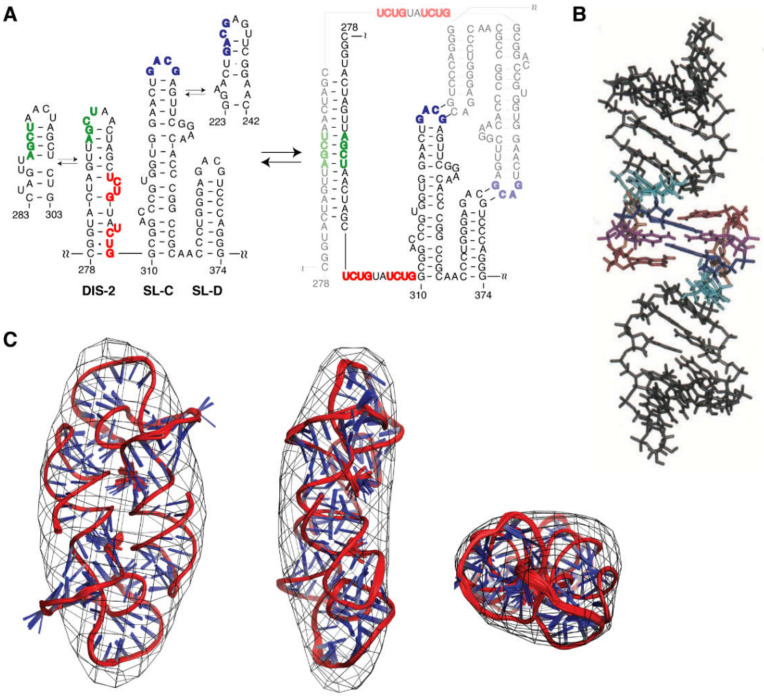
Dimerization-dependent structure of the MoMuLV minimal packaging signal. (**A**) Left: Secondary structure of monomeric mΨ and the base pairings observed at equilibrium for DIS-2 and SL-C. Right: Base pairings in the dimeric form of mΨ. The palindromic sequences required for dimer formation are shown in green (DIS-2) and blue (SL-C and SL-D). NC-binding UCUG elements are shown in red. (**B**) The NMR structure of the 18-nt SL-D kissing duplex. (**C**) Superposition of the cryo-electron tomography densities and the NMR ensemble structures of [Ψ^CD^]_2_. Panels (**A**,**C**) reproduced from [315], and panel (**B**) from [316], with permission.

Mutagenesis, chemical accessibility probing, and computational analysis indicated that the Ψ region folds into several stem-loops, which are further condensed by long-range interactions [58,65,305,306,307,308,309,310]. Deletion of conserved residues predicted to form adjacent stem-loop structures (SL-C and SL-D; Figure 13), eliminated the RNA packaging [309,311], and heterologous RNAs containing SL-C, SL-D, and a portion of an upstream pseudopalindrome (SL-B) were efficiently packaged into virions [58] (although SL-C and SL-D alone were insufficient to promote packaging [312]). Native gel electrophoresis and ITC studies also showed that NC binds with a high affinity to an RNA fragment containing all three stem-loops (K_d_ ~132 nM), but interacts weakly with constructs comprising only one or two stem-loops [313,314]. Thus, this region of the MoMuLV genome [stem-loops DIS-2 (SL-B), SL-C and SL-D] is commonly referred to as the core encapsidation signal (Ψ^CES^).

The MoMuLV Ψ^CES^ RNA readily forms dimers under physiological-like ionic strength conditions (as does the entire leader) and undergoes conformational rearrangement upon dimerization [310]. Specifically, DIS-2 (also called SL-B), and an upstream pseudopalindrome called DIS-1 (or SL-A), undergo a register shift in base-pairing [66,310,315,317] upon formation of extended intermolecular duplexes [65,317,318,319,320]; Figure 13. Tinoco et al. were the first to show that SL-D is capable of forming a kissing dimer stabilized by only two intermolecular G-C base pairs [316] (Figure 13B), which was confirmed by subsequent NMR studies [314,315]. The kissing interaction was also characterized by nonequilibrium all-atom molecular dynamics simulation [321]. The tertiary structure of the native SL-CD dimer was determined by NMR using NOE and RDC restraints, and the overall shape of the NMR structure was confirmed by cryo-EM [315]; Figure 13C.

**Figure 14:**
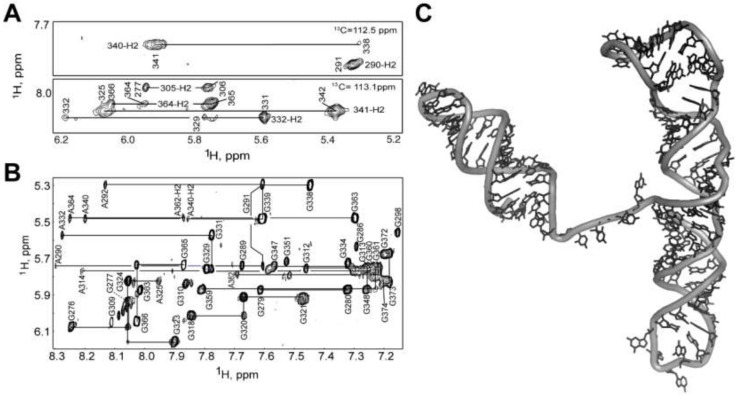
Improved NMR spectral quality afforded by ^2^H-edited NMR in the studies of MoMuLV minimal packaging signal, with loop residues engineered to prevent dimerization (mΨ^CES^). (**A**) Representative strips from 3D ^1^H-^13^C correlated with the NOESY spectra obtained for ^13^C-labeled mΨ^CES^. (**B**) Portion of a 2D NOESY spectrum obtained for an mΨ^CES^ sample containing protonated guanosines and with all other predeuterated nucleotides (breakthrough signals from incomplete deuteration of adenosines are also visible). Linewidths are significantly narrower in the ^2^H-edited spectra. (**C**) 3D NMR structure of mΨ^CES^. Adapted from [322], with permission.

The 3D structure of a mΨ RNA with mutated loops to prevent dimerization was determined by a ^2^H- and ^13^C-edited NMR approach [322]; Figure 14. The improved ^1^H NMR linewidths and spectral quality were readily apparent upon comparison of the ^2^H- and ^13^C-edited NMR spectra; Figure 14A,B. Stem-loop SL-B contains five mismatched base-pairs and connects to SL-C via a 4-nt linker. SL-C contains an unusual base-triple platform, and a novel “A-minor K-turn.” SL-D adopts a classical A helical structure and is coaxially stacked with SL-C; Figure 14C. The NMR structure differs significantly from the models proposed on the basis of structural probing, using selective 2′-hydroxyl acylation analyzed by primer extension (SHAPE) technology [323], in which the DIS-2 hairpin was proposed to adopt an alternative fold and a large portion of the RNA (residues 283–303) was suggested to be unstructured [324].

#### 3.2.2. Rous Sarcoma Virus (RSV)

RSV is an alpha-retrovirus and was the first oncovirus described that could induce connective tissue tumors in chicken [325]. Unlike other retroviruses, the entire 5′-leader sequence (1-397 nt) of RSV is located upstream of SD and is thus included in all spliced and unspliced viral RNAs [326]. Like other retroviruses, RSV encodes a conserved 5′-leader with PBS, DIS, ψ, and AUG structural elements. The 5′-leaders of avian retroviruses also contain three short open reading frames (ORFs) that were proposed to regulate ribosome scanning [327,328]. One of the ORFs (ORF3) resides within the packaging signal [61]. Some mutagenesis studies suggested that the translation of ORF3 might directly or indirectly increase the packaging efficiency [329,330,331]. A study that competitively assessed Gag- versus ribosome-binding suggested that translation of ORF3 is not required for packaging, but is instead critical for maintaining the balance between translation and packaging [328].

The packaging sequence of RSV was initially discovered by spontaneous deletions near the 5′ end of viral genome, which lead to poor viral infectivity and deficient genome packaging [332,333,334,335,336,337]. All of these deletions contain a 31-nt region between the PBS and SD sequences [338]. A series of studies indicated that the region between the PBS and SD is required for genome packaging and is sufficient to direct the encapsidation of heterogenous RNAs into virus particles [59,60,332,334,335,337,338,339,340], while residues upstream [341,342] and downstream [60,339,343] of this region have little or no effect on packaging efficiency. The packaging signal was further refined to a 160-nt region called MΨ (nucleotides 156–315) [61,63]. Unspliced heterologous RNAs containing MΨ are packaged only about three-fold less efficiently than the wild-type genomic RNA [60,63]. A phylogenetic sequence alignment study predicted the secondary structure of the first 499 nucleotides in 13 strains of avian retroviruses, which consists of leader loops (L1-5), the PBS loop, the O3 loop located just downstream of the ORF3, and G1-3 loops located in the *gag* coding region [327]. Most of these stem-loop structures are supported by SHAPE experiments [344]. Computational models of MΨ in different strains of avian sarcoma leukosis virus (ASLV) also predicted two major stem-loops: O3 and L3 [61,62,327].

The O3 stem was found to be essential for genome packaging [62,327,345]. Mutations that disrupted the base-pairing of the stem, reduced packaging by 100-fold, compared to wild-type levels [62,327], whereas restoration of the base-pairing restored packaging efficiency [345]. The O3 stem-loop alone was sufficient to direct RNA packaging with efficiency equal to that of MΨ, thus, defining the minimal packaging determinant of the RSV genome (µΨ) [64]. The O3 stem-loop was predicted to consist of three minor stem-loops (O3-SLA, B, and C). Mutations designed to disrupt the minor loop structures greatly reduced packaging, while mutants that maintain RNA structure showed no difference or showed a slightly reduced packaging efficiency compared to the wild-type RNA [61,64], indicating that the structure rather than the sequence of O3 is crucial for genome packaging. Native polyacrylamide gel electrophoresis (PAGE), ITC, and NMR studies revealed that O3 interacts with NC with very high affinity (K_d_ =1.9 nM). However, isolated stem-loops and multi-stem-loop-fragments bind weakly to NC [346]. These findings set the stage for NMR-based 3D structural studies of the NC: µΨ complex [112] (see below).

It remains to be determined if or how RSV genome dimerization is coupled with packaging [347,348,349,350]. The palindromic sequence in the L3 loop [63,191,351], as well as palindromes in upstream [191] and downstream [347,352] sequences, were proposed to play roles in ALSV dimerization. Mutagenesis studies suggested that the L3 loop might indirectly contribute to packaging, by stabilizing the structure of the packaging signal [62,63]. A SHAPE experiment of the first 636-nt of RSV, mapped out the secondary structure of the entire leader [344]. In this study, L3, and O3-SLA were shown to be required for dimerization, and mutations in the loops of any of these elements abolished dimerization, but did not lead to significant reductions in viral replication. These findings suggested that the dimerization observed in in vitro studies might not be biologically relevant [344]. It is also interesting that the RSV packaging signal is present in both the spliced and unspliced 5′-UTR, even though the unspliced transcripts are preferentially packaged (200-fold enhancement compared with spliced transcripts) [31]. The mechanism through which RSV distinguishes between spliced and unspliced transcripts remains to be understood. One possibility is that the higher order structure of the leader in the unspliced RNA might be different from that in spliced RNAs, which favors a Gag-binding conformation [15].

## 4. NC–RNA Interactions

### 4.1. HIV-1

Initial characterizations of the nucleic acid binding activity of HIV-1 NC were performed mainly using deoxyribonucleotides or short polypeptides consisting of one zinc knuckle domain [82,107,111,353,354,355]. NMR titration experiments revealed that the hydrophobic residues Phe16 and Ile24 of the N-terminal zinc knuckle domain (NC-F1) were directly involved in the interactions with single-stranded DNA substrates [107,353,354]. Disruption of the zinc knuckle structure by the addition of a zinc chelating agent (ethylenediaminetetraacetic acid) abolished these interactions [107]. Stacking interactions between NC-F1 aromatic side-chains and nucleic acid bases were suggested to be important for binding [353,355]. Early fluorescent studies showed that NC binding is sequence-specific [82]. NMR titration experiments using DNA oligonucleotides with varied sequences, further revealed that the tight binding to HIV-1 NC-F1 requires the presence of at least one guanosine residue [107]. Two-dimensional NOESY-data-facilitated structural modeling revealed that the packaging site analog, d(ACGCC), binds within the hydrophobic cleft on the NC-F1 surface, and the complex is further stabilized by multiple intermolecular hydrogen bonds involving the guanosine base [107].

**Figure 15:**
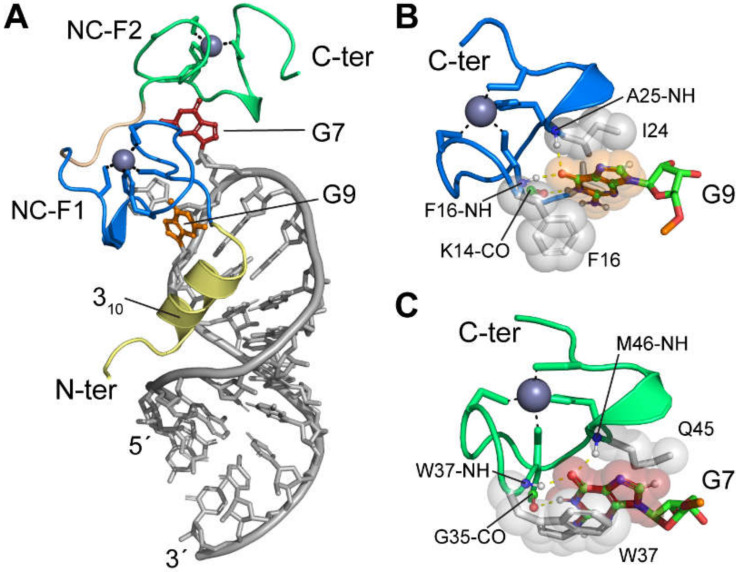
NMR structure of HIV-1_NL4-3_ NC bound to the GGAG loop region of the Ψ-hairpin stem-loop [109]. (**A**) Overall view showing the relative orientation of the RNA (gray) relative to the 3_10_-helix (yellow), N-terminal zinc knuckle (F1, blue), and C-terminal zinc knuckle (F2, green). Guanosines G7 and G9 are shown in red and orange, respectively. (**B**) Interactions between G9 and NC-F1. Hydrogen bonds are depicted as yellow dash lines and hydrophobic side chains shown as spheres. (**C**) Interactions between G7 and NC-F2.

The three-dimensional structure of HIV-1 NC bound to a 20-residue RNA, containing the apical loop of the Ψ-hairpin (originally called “SL3”), was determined by heteronuclear NMR spectroscopy [109]. The specific recognition was mainly mediated by the interactions between the G9 and G7 nucleotide bases of the G6-G7-A8-G9 tetraloop and the hydrophobic clefts of the N- and C-terminal zinc knuckles, respectively (Figure 15A). In the NC-Ψ-hairpin complex structure, the base of G9 is sequestered into the NC-F1 hydrophobic cleft, and directly contacts the bulky side chains of Val13, Phe16, and Ile24; Figure 15B. G9 is further stabilized in the NC-F1 hydrophobic cleft through specific hydrogen bonds between G9-O6 and the backbone NH groups of Phe16 and Ala25, and between G9-H1 and the backbone carbonyl group of Lys14; Figure 15B. G7 interacts with the C-terminal zinc knuckle (NC-F2) hydrophobic cleft in a very similar manner with the nucleotide base packed against the side chains of conservatively substituted Trp37 and Gln45. Additionally, the G7-O6 forms hydrogen bonds with the backbone NH groups of Trp37 and Met46, and G7-H1 forms a hydrogen bond with Gly35-CO; Figure 15C. Extensive non-specific electrostatic interactions also contribute to the tight binding between NC and the Ψ-hairpin [109]. The HIV-1 NC also exhibits structural rearrangements upon binding to the Ψ-hairpin, including the formation of a 3_10_ helix (Lys3 to Arg10) and RNA binding-induced contacts between the two zinc knuckle domains [109], which are hallmarks of adaptive binding.

The 3D structure of HIV-1 NC bound to a 19-residue SD-hairpin was also determined by solution NMR [110]. Similar to the specific interactions observed in the NC-Ψ-hairpin structure, the hydrophobic clefts of the N- and C-terminal zinc knuckle domains bind to exposed guanosine bases G11 and G9 of the G8-G9-U10-G11 tetraloop of SL2, respectively. The G11 base is sandwiched between the hydrophobic side chains of Phe16 and Ile24 and stabilized by the hydrogen bonds between G11-O6 and the backbone amide groups of Phe16 and Ala25, as well as by a hydrogen bond from G11-H1 to the backbone carbonyl of Lys14. In the NC-F2 hydrophobic cleft, the G9 nucleobase packs against the side chains of Trp37 and Gln45, and is stabilized by hydrogen bonding from G9-O6 to the backbone amide groups of Trp37 and Met46, and from G9-H1 to the Gly35 backbone carbonyl. Thus, the NC zinc knuckles bind to exposed guanosines of the Ψ-hairpin and SD-hairpin RNAs, in a similar manner. Residues Lys3 to Arg10 also form a 3_10_ helix, upon binding to SD-hairpin, but form different protein-RNA interactions, compared to that of the NC-Ψ-hairpin structure [109,110]. NMR NOE analysis also reveals that the RNA-induced contacts between the NC-F1 and NC-F2 zinc knuckles differ among the SD-hairpin and Ψ-hairpin NC complexes [110]. Thus, it is conceivable that HIV-1 NC exhibits inherent structural flexibility, which allows NC to adaptively bind to different RNA targets with high-affinity.

In addition to the loop region of RNA hairpins, the three-dimensional structure of NC bound to a single-stranded U5 derivative (5′ -UGUGCCCUUCU-3′) was also determined using solution NMR [114]. The two guanosines of this RNA form very similar interactions with the NC-F1 and NC-F2 hydrophobic clefts in the same polarity, as reported for the NC-Ψ-hairpin and NC–SD–hairpin complexes (NC-F1 binding to 3′-guanosine, NC-F2 binding to 5′-guanosine) [114]. Extensive biochemical data demonstrated that the HIV-1 packaging signal contains more than two dozen NC binding sites, with varied affinities and different functional importance [40,41,71]. In particular, NMR-detected NC binding, in conjunction with ITC studies reveal that the [UUUU]:[GGAG] stem region of the Ψ-hairpin contains two very high-affinity binding sites (K_d_ ~40 nM, compared with K_d_ ~ 300 nM for the Ψ-hairpin apical loop, under physiologic-like ionic strength) [236]. The structural lability of this [UUUU]:[GGAG] helical region is shown to be required for both tight NC binding in vitro and efficient RNA packaging in transfected cells [236]. However, the molecular mechanism for such high-affinity binding remains unknown, and additional studies of NC bound to other RNA targets in the HIV-1 dimeric 5′-leader are clearly warranted.

### 4.2. Other Retroviruses

#### 4.2.1. MoMuLV

Initial NMR studies of MoMuLV NC-nucleic acid interactions were conducted with a pentanucleotide, d(ACGCC), which was previously shown to bind to a HIV-1 zinc knuckle peptide [107] and to HIV-1 NC [111,356]. ^1^H-NMR studies of the complex identified a cluster of hydrophobic residues on the surface of the MoMuLV zinc knuckle that form a binding pocket for a guanosine base (Leu21, Ala27, Ala36). ^31^P NMR experiments revealed significant chemical shift changes for phosphodiester groups between G3 and C4, and between A1 and C2. These results revealed that the MoMuLV zinc knuckle binds exposed guanosines bases in a manner similar to that observed for HIV-1 zinc knuckles.

Unexpectedly, MoMuLV NC did not exhibit significant affinity for any isolated Ψ^CES^ hairpins (DIS-2, SL-C or SL-D), nor to the SL-CD tandem stem-loop RNA [314]. Instead, the protein only bound tightly to RNA constructs containing all three stem-loops (the intact Ψ^CES^), and the binding required (and was shown to induce) RNA dimerization [314]. These data supported a mechanism in which MoMuLV NC targets a binding site that requires the presence of all three hairpins (SL-BCD, Ψ^CES^) [314].

**Figure 16:**
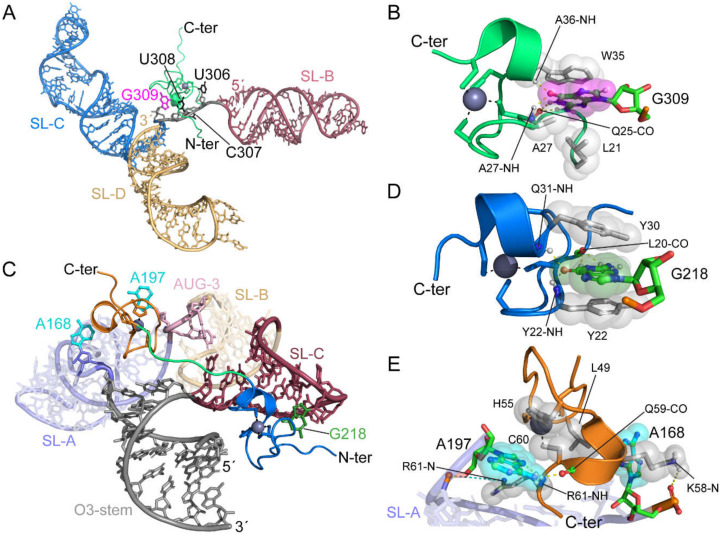
Complex structures of MoMuLV and RSV NC bound to the recognition elements in their respective packaging signals (reported in [66] and [112], respectively). (**A**) MoMuLV NC-UCUG complex structure (PDB ID: 1U6P). SL-B, SL-C, and SL-D are colored in red, blue, and gold, respectively. The UCUG linker and binding site is colored gray with G309 in magenta. The zinc knuckle domain of the MoMuLV NC is shown in green, with the black dashes representing the coordination of Zn. (**B**) The direct interaction between the MoMuLV NC and G309. Hydrogen bonds are shown as yellow dashes and the gray spheres represent hydrophobic interactions. (**C**) RSV NC bound to the µΨ packaging signal (PDB ID: 2IHX). The O3-stem, SL-A, AUG-3 linker, SL-B, and SL-C are colored gray, purple, pink, gold, and red, respectively. The N-terminal zinc knuckle, linker residues, and C-terminal zinc knuckle are colored blue, green, and orange, respectively. G218 nucleobase is colored green. A168 and A197 nucleobases are colored cyan. (**D**) The N-terminal zinc knuckle of the RSV NC interacting with G218. (**E**) The C-terminal zinc knuckle interacting with A168 and A197. Salt bridge interactions are depicted as cyan-colored dashes.

To facilitate 3D structural studies, a Ψ^CES^ construct, with loop residue mutations designed to prevent dimerization, while retaining the internal base pairing of the dimer (SL-Bm-Cm-Dm, mΨ^CES^) was examined [66,314]. These studies revealed that the single zinc knuckle domain of MoMuLV NC, binds specifically to the UCUG linker that connects the DIS-2 helix and the SL-C hairpin (residues U306–G309); Figure 16A. The bases of nucleotides U306, C307, and U308 pack against a hydrophobic surface comprising the side chains of Leu21, Ala27, and Tyr28, and binding is further stabilized by a number of direct or water-mediated salt bridges and hydrogen bonds [66]. The G309 base fits into a hydrophobic cleft, lined by the side chains of Leu21, Ala27, Trp35, and Ala36, and is anchored by the following hydrogen bonds—G309-O6–Ala27-NH, G309-O6–Ala36-NH, G309-N1H–Gln25-O, G309-NH21–Gln25-CO; Figure 16B [66]. These hydrophobic and hydrogen bonding interactions are similar to those observed for the guanosine-binding zinc knuckles of HIV-1 NC.

Chemical accessibility experiments conducted with a larger portion of the MoMuLV 5′-leader indicated that multiple UCUG sequences become exposed and accessible, following RNA dimerization, suggesting the presence of additional NC-binding sites for genome recognition and packaging [308,310]. Short oligoribonucleotides were used in a combination of NMR and ITC studies, to characterize the effect of the residues flanking the guanosine on NC-binding and to determine the binding affinity of NC to the other possible recognition elements. Three specific oligoribonucleotides that represented the proposed binding sites were of specific interest—AACAGU, UUUUGCU, and CCUCCGU. Based on 2D-NOESY data, the guanosine at position *i* gave rise to intermolecular NOEs between G_i_-H8 and Trp35 aromatic, Ala27 methyl, Ala36 methyl, and Leu21 side-chain protons, and between G_i_-H1′ and Leu21, Trp35, and Arg23 side-chain protons [113]. Additional NOEs were observed between the nucleobase and ribose protons of residues at positions *i*-1, *i*-2, and *i*-3, and the side-chain protons of Ala27, Leu21, Arg18, Tyr28, Ala36, Lys42, and Lys30 [113]. These findings suggest a form of cooperative recognition mechanism where the initial NC-binding might promote RNA dimerization and concomitant exposure of additional NC-binding sites.

#### 4.2.2. Rous Sarcoma Virus

The structure of the RSV NC bound to the minimal packaging signal, µΨ, is also solved by NMR. Unlike other retroviruses, the 5′ UTR of alpha-retroviruses contain three distinct open reading frames or AUG motifs—AUG-1, AUG-2, and AUG-3, and there is controversy over their functional differences [328,329,330,331]. The proposed structure of the minimal packaging signal consists of a central stem, O3 stem, with a 3-way junction between the three stem-loops—SL-A, SL-B, and SL-C where SL-A and SL-B are linked via AUG-3; Figure 16C [64,112] (see above). NMR studies of the NC:µΨ complex suggest that the AUG-3 in the µΨ region is a binding site for NC [112,328,357]. The RSV NC protein has distinct N-terminal and C-terminal zinc knuckle motifs that bind viral RNA through different mechanisms.

NMR analysis showed that the SL-C tetraloop gave rise to distinct NOEs with the aromatic residues in the N-terminal zinc knuckle [112]. Similar to the zinc knuckle domains of HIV-1 and MoMuLV [9,92], the N-terminal zinc knuckle of the RSV NC contains a hydrophobic pocket defined by Tyr22, Tyr30, Leu20, and Gln30, which fits the nucleobase of G218 [112]. The complex is further stabilized via intermolecular hydrogen bonding between backbone amides and oxygens—G218-O6–Tyr22-NH, G218-O6–Gln31-NH, G218-N1H–Leu20-O, and G218-NH21–Leu20-CO; Figure 16D. NOE analyses also indicated that linker residues U224, A225, and G226 fold into the minor groove of SL-C [112].

The C-terminal zinc knuckle structure lacked a hydrophobic pocket and did not give rise to NOEs to guanosine residues, suggesting that it functions differently than other characterized zinc knuckle domains. However, there were NOEs to the linker residues adjacent to the 5′ and 3′ bases of SL-A [112]. On the 3′-end, the exocyclic amino group and N7 of A197 (the adenosine in AUG-3) formed hydrogen bonds with the carbonyl group of Gln59 and the amide proton of Arg61, respectively. The nucleobase of A197 is positioned against His55, Cys60, and Arg61, and is further stabilized by a salt-bridge between the 5′-phosphate of A197 and Arg61; Figure 16E. On the 5′ base of SL-A, the nucleobase of A168 is stacked against Leu49, Cys60, and Lys58 side-chains, and it is further stabilized via hydrogen bonding between the A168 phosphate oxygen and the Lys58 side-chain amino group; Figure 16E [112]. The significance of these interactions was further supported by in vivo mutagenesis studies, where inserting a non-native GAGA SL-C tetraloop or substituting the AUG-3 linker residues significantly reduced viral infectivity [112].

### 4.3. Chaperone Activity of NC

In addition to its role in genome packaging, the NC domain of Gag (or the mature NC protein) functions as an RNA chaperone to catalyze conformational rearrangements [358]. NC stimulates reverse transcription by annealing the tRNA^Lys3^ primer to the primer binding site (PBS) [359,360], promotes the processing of reverse transcriptase by reducing polymerization pausing [361,362], and increases the efficiency of both minus- and plus-strand transfers during reverse transcription [363,364,365,366,367,368,369,370]. NC chaperone activity was also proposed to help catalyze the kissing to extended dimer conversion (see above). The chaperone property of retroviral NC appears to rely on both the highly basic N-terminal residues and the zinc knuckle structures of NC, which involves weak and non-specific NC–RNA interactions [214,371,372]. NMR studies showed that NC performs its chaperone activity by lowering the energy barrier required to break base pairs or by facilitating the formation of new base-pairs [169,358,373,374,375,376,377,378].

## 5. Inhibition of Genome Packaging

Efforts to develop inhibitors of HIV-1 genome packaging involved approaches that targeted both the NC domain of Gag and the RNA packaging signal. Small molecules that bound to both viral targets and interfered with NC-RNA binding in vitro, were identified. Although these compounds exhibited antiviral activity in cell culture assays, none exhibited the appropriate efficacy and toxicity required for advancement to the clinic. The current progress is summarized below.

### 5.1. Inhibitors that Target NC

**Figure 17:**
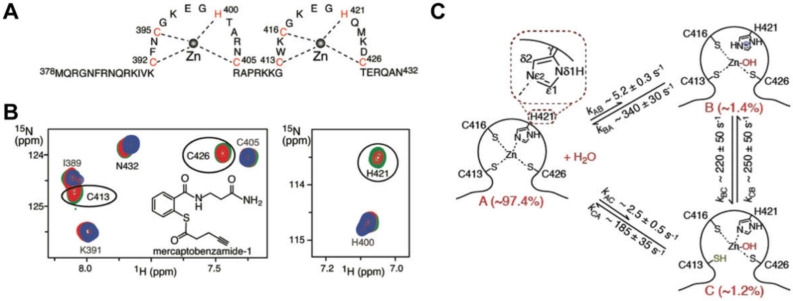
(**A**) NC treated with mercaptobenzamide-1 targets the sparsely populated states of the C-terminal zinc knuckle of NC. (**B**) ^1^H-^15^N spectrum of NC alone (green) treated with 20-fold molar excess of mercaptobenzamide-1. Three-hour treatment (red) and 6-h treatment (blue) of merceptaobenzamide-1 (Black circles highlight the C-terminal zinc knuckle cross-peaks). (**B**,**C**) ^15^N-CPMG relaxation dispersion (**B**) and CEST experiments reveal the sparsely populated states (**C**) of the C-terminal zinc knuckle of NC. Adapted from [384], with permission.

Current anti-retroviral therapies involve a combination of drugs that suppress HIV-1 replication, but resistance can still occur, and this life-long treatment regimen can have adverse side effects. Therefore, there is a need to develop antivirals that target different components of HIV that are essential for replication. The zinc knuckles of NC are essential for replication and, therefore, are attractive potential targets. One class of antiviral agents was shown by NMR to function by a novel “zinc ejection” mechanism, in which the cysteine sulfur atoms that are required for binding zinc and maintaining proper folding are selectively oxidized [379,380,381,382,383]. Mercaptobenzamide thioesters initially oxidize a specific cysteine of the C-terminal zinc knuckle; Figure 17. NMR chemical exchange saturation transfer (CEST) and relaxation experiments showed that the C-terminal zinc knuckle undergoes conformational exchange between one major species and two minor species, which likely explains the enhanced reactivity of the C-terminal zinc knuckle; Figure 17 [384].

**Figure 18:**
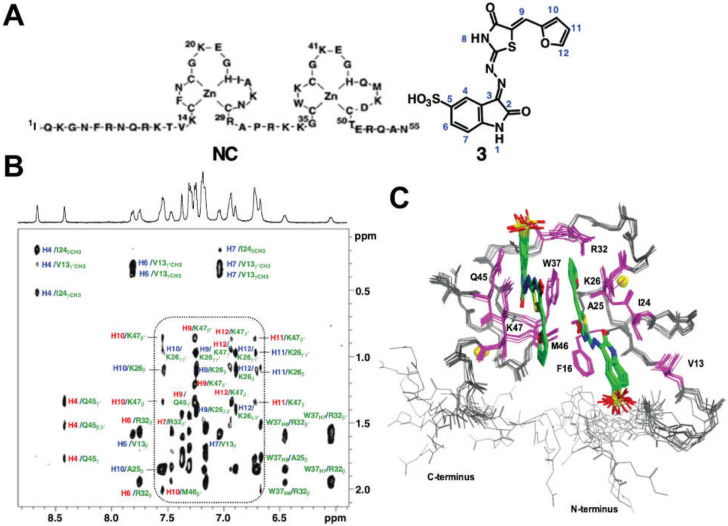
Small molecule inhibitors of NC-oligonucleotide binding. (**A**) NC protein and small molecule (Inhibitor-3) used for structural studies. (**B**) 2D NOESY data for the NC:Inhibitor-3 complex. (**C**) NMR structure of the NC:Inhibitor-3 complex (carbon atoms in green). Adapted from [385], with permission.

More recently, a series of compounds were identified that could block interactions between NC and TG-rich deoxynucleotides. NMR-based structural studies revealed that two inhibitor molecules interact with the N-terminal and C-terminal zinc knuckles by mimicking exposed guanosine residues [385] (Figure 18), which are known to be important for binding nucleic acids [66,107,109,110].

### 5.2. Inhibitors that Target the RNA Packaging Signal

**Figure 19:**
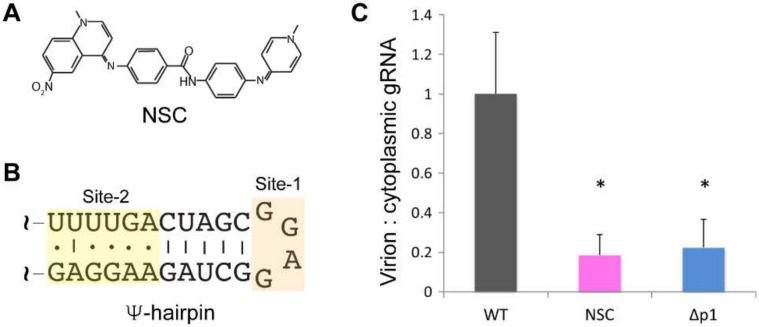
Small molecule inhibitor of HIV-1 genome packaging. (**A**) Structure of packaging inhibitor NSC. (**B**) Binding sites of NSC in the Ψ-hairpin of the HIV-1 packaging signal. (**C**) NSC treatment reduces HIV-1 selective genome packaging. Asterisks represent statistically significant from wild-type (WT) by Student’s *t* test, *p* < 0.05. Panel (**C**) reproduced from [389], with permission.

In addition to the viral proteins, the highly conserved and structured regions of the RNA genome are also attractive therapeutic targets, as exemplified by the Ψ-hairpin of the HIV-1 RNA packaging signal [386]. Multiple small molecules were identified to inhibit NC-Ψ interactions [387,388]. Among them, a quinolinium derivative NSC260594 (NSC, Figure 19A), was shown to be a specific HIV-1 RNA packaging inhibitor [389] and exhibited potent antiviral activity [388,389]. NSC treatment caused a similar packaging defect as that of the Δp1 mutation—a 19 nt deletion that historically led to the identification of the Ψ-hairpin as a major packaging determinant; Figure 19C [228,389]. NSC was initially identified as an inhibitor of the interaction between Gag and a 20-nt construct containing the upper stem and the apical tetraloop of the Ψ-hairpin [388]. In the same study, ^1^H NMR spectroscopy showed that NSC specifically bound to the GGAG tetraloop (site-1 in Figure 19B). However, a recent NMR study demonstrated that NSC targeted the structurally labile [UUUU]:[GGAG] stem region of the Ψ-hairpin, in the context of a larger construct of the HIV-1 packaging signal (site-2 in Figure 19B) [236]. Using the full-length HIV-1 5′-leader, SHAPE analysis suggested that the function of NSC is to stabilize the Ψ-hairpin and subsequently stabilize a larger region of the RNA packaging signal [389]. These studies further confirmed the critical function of the [UUUU]:[GGAG] region of the Ψ-hairpin in HIV-1 selective genome packaging, which could serve as a novel specific therapeutic target.

In addition to small molecule inhibitors, ^1^H NMR analysis showed that polypeptides rich in tryptophan also interact with the GGAG tetraloop of the Ψ-hairpin, and possibly block NC binding [390]. The DIS region might also be targeted, since dimerization is a prerequisite for retroviral genome packaging [391].

## 6. NMR-Based Hybrid Approaches

Many of the challenges associated with NMR studies of large RNAs and protein-RNA complexes are natural targets for hybrid approaches that provide complementary structural information. Commonly employed NMR methods provide high-resolution local structural information but do not inform on overall molecular shape. In contrast, methodologies such as SAXS and cryo-EM, provide information on global structure and overall shape, but typically do not afford information regarding high-resolution local structural features. Hybrid approaches that combine NMR restraints with global shape restraints derived from SAXS or cryo-EM, offer an attractive approach for the study of large RNAs and protein:RNA systems that are difficult to study by any one method.

SAXS has many advantages in this regard, because (i) small-angle scattering profiles do not require sample crystallization, (ii) there are no size limitations, (ii) the electron-rich backbone of RNA scatters X-rays strongly, (iii) the 3D RNA structures are typically non-spherical, and (iv) procedures for implementing sparse NMR restraints and SAXS data could be accomplished with existing NMR structure refinement software packages [392,393,394,395,396]. A disadvantage of SAXS is that data analysis can be complicated if the sample exists as a mixture of multiple conformers or contains even small amounts of higher-order species. An attempt to use SAXS to analyze the structure of a tandem hairpin RNA structure that is important for MoMuLV genome packaging was unsuccessful due to the fact that the RNA existed in solution as a mixture of monomeric, dimeric, and higher-order multimeric species, each of which was directly detectable in a single sample, through cryo-EM [315]. Although there are presently no examples of hybrid SAXS/NMR methods applied to the studies of retroviral genome packaging, these approaches provided important insights into HIV-1 RNA structures that contribute to transcriptional activation [274], splicing [397], and nuclear export [398]. For example, Tolbert and co-workers used a hybrid SAXS/NMR approach to determine the structure adopted by residues of a phylogenetically conserved intron splice silencer stem-loop. A handful of cellular RNAs were also examined by this approach (for examples, see [394,399,400,401,402]).

Cryo-EM is another complementary technique traditionally used to determine the tertiary structures of large protein complexes [403,404]. Unlike SAXS and NMR, samples are frozen in a thin layer of vitrified ice and density maps generated from the scattering data were obtained for a large number of individual particles, thereby producing a 3D envelope for an ensemble average of particles [405]. A particular advantage of cryo-EM is that densities for individual molecules that exist in different conformations or oligomerization states can be “binned,” enabling studies of samples containing structurally heterogeneous molecules. Resolution is limited by several factors, including flexibility, symmetry, and size [404]. Cryo-EM is historically used to study the structures of large macromolecular assemblies, and it was initially surprising that high-quality electron density maps could be obtained for the structures of RNAs as small as the SL-C/SL-D tandem hairpin of the MoMuLV packaging signal (see Figure 13, above) [315]. At the time, this RNA was simultaneously among the largest RNAs to be structurally characterized by NMR, and the smallest to be characterized by cryo-EM [315]. These studies revealed that the helical regions of the tandem hairpin pack side-by-side, a finding that was inferred by the NMR-directed calculations but not experimentally verifiable using the NMR methods employed. Perhaps the most important point of this early work was that the low-resolution global structural information obtainable by cryo-EM was highly complementary to the high-resolution local structural information readily obtainable by NMR [315].

The combination of NMR and cryo-EM was further utilized in the structural characterization of the extended duplex form of the 30 kDa HIV-1 DIS [204]; Figure 6E. The cryo-EM map, refined to 9 Å resolution, revealed the major groove of the RNA and the presence of an overall super-helical twist. The cryo-EM data also revealed bulges in the electron density maps, indicative of bulged nucleotides. The combination of NMR and cryo-EM in this study clearly demonstrated the utility of merging global and local restraints in structure determination [204].

## 7. Summary and Future Directions

During the late 1980’s and early 90’s, NMR was used to study relatively small retroviral proteins and RNAs were believed to be important for packaging. These studies provided insights into the role of zinc in viral replication, characterized the structure of NC, and its intrinsic CCHC zinc knuckle domains, and compared and contrasted the NC domains of different retroviruses. These studies were complemented by studies with small nucleic acids, which identified sequences that bind the NC zinc knuckles with high affinity, established the mechanism of DIS-promoted RNA dimerization, and led to the structural characterization of nucleic acid complexes with isolated zinc knuckles and intact NC proteins. NMR studies also provided insights into the structures of other regions of the 5′-leader that are important for viral replication, and led to the identification of novel reagents and mechanisms for inhibiting viral replication by promoting zinc ejection from the CCHC zinc knuckles or binding competitively to NC or to RNA elements within the 5′-leader. In addition, NMR studies of a minimal packaging signal from the MoMuLV 5′-leader, led to the proposal for a dimerization-dependent RNA structural switch packaging mechanism, in which nucleotides important for NC binding are sequestered by base pairing in the monomeric form of the RNA and become exposed to promote NC-binding upon dimerization. It remains to be determined if MoMuLV or other retroviruses utilizes heterogeneous transcriptional start site usage as a means of controlling transcript dimerization, as appears to be the case for HIV-1. Interestingly, more than 40 years ago, Levin and Rosenak proposed that MuLV-producing cells likely contain two non-interconverting pools of viral transcripts that function separately as viral mRNA and gRNA, based on the sensitivity of gRNA incorporation into virions produced in the presence of the transcription inhibitor, actinomycin D [406], and more recently Mougel et al. showed that nuclear export of MuLV gRNA and mRNAs occurs via different pathways [407]. Although HIV evolved a Rev-dependent mechanism to promote nuclear export [408], it remains plausible that the evolutionarily distant HIV and MoMuLV retroviruses could both utilize heterogenous transcriptional start site usage as a means of producing distinct pools of messenger and genomic RNA transcripts.

RNA dimerization-dependent packaging mechanisms were proposed for HIV, based primarily on biochemical and nucleotide accessibility mapping studies of in vitro transcribed RNAs, but applications of NMR to test structural and mechanistic hypotheses were not performed until the past decade or so, due to the large size of the HIV 5′-leader (~350 nucleotides). The first reported NMR studies of the intact HIV-1 5′-leader were performed using both chemical ligation of differentially labeled (^13^C-labeled and un-labeled) fragments and nucleotide specific ^2^H editing. Both strategies aimed to enhance spectral sensitivity and resolution and enable detection of NMR chemical shifts and NOE signals for the specific nucleotides of interest [409]. Although the ^1^H-^13^C signals were detectable for the regions of the leader undergoing rapid rotational motions (e.g., the 3′-terminal AUG hairpin), they could not be detected for residues in larger, more globular regions of the RNA undergoing more restricted rotational motions (e.g., the AUG residues when base paired with the upstream U5 element). Thus, although the ^1^H-^13^C correlated NMR methods that are widely used to study protein structure were able to characterize smaller, structurally independent regions of the leader, they had a limited utility for the characterization of large globular regions. For example, ^1^H-^13^C correlated NMR methods showed that the AUG residues adopt a hairpin in the monomer and an alternate (undetermined) structure in the dimer, likely due to their participation in a larger, globular structure in the dimer.

In contrast, the signals observed for the adenosine-H2 protons in highly deuterated samples exhibited good resolution and sensitivity for a majority of the adenosines, enabling a more complete analysis of the RNA structure involving these residues (and nearby protonated nucleotides). Initial studies focused on the non-capped 2G HIV-1_NL4-3_ leader that adopts a stable dimer structure under physiological-like conditions, and the NMR studies confirmed several aspects of secondary structures that were proposed on the basis of chemical probing and phylogenetic analyses. For example, signals in the ^2^H-edited NOESY NMR spectra obtained for the dimeric leader, were consistent with a structure in which the residues of TAR and polyA form hairpins and residues of AUG base pair, with an upstream U5 segment.

Attempts to study the monomeric form of the 2G HIV-1_NL4-3_ leader were confounded by the inability to obtain homogeneous samples of monomeric species, at the high concentrations required for NMR measurements. Nevertheless, studies of samples with measurable populations of the monomer revealed that residues of AUG form a hairpin, and residues of the DIS palindrome form base pairs with U5, in the monomeric form of the HIV-1_NL4-3_ leader. Based on the observation of A-H2 signals that are unambiguously diagnostic of the DIS hairpin, those studies suggested that the DIS retains its hairpin structure in the monomer. Subsequent studies shifted focus to the leader of HIV-1_MAL_ because the samples containing higher percentages of the monomer could be obtained. These studies clearly showed that the DIS does not adopt a hairpin structure in the monomer, and it now appears that the DIS hairpin signals observed in the HIV-1_NL4-3_ spectra were associated with an equilibrium presence of the dimeric species. The NMR data clearly showed that the monomeric form of the HIV-1_MAL_ leader adopted a structure in which the DIS palindrome formed base pairs with U5, as observed by NMR for the NL4-3 leader, and the remaining residues form long-range base pairs with residues of the upstream polyA and U5 elements. This topology has similarities to the “Long Distance Interactive” (LDI) model proposed by Abbink and Berkhout (although aspects of the LDI and NMR-derived models differ). NMR also helped shed light on the factors that modulate RNA dimerization and thereby modulate the transcript function and fate. 5′-Capped transcripts predominantly begin with either one or three guanosines, resulting from “twinned” start site usage. Transcripts that begin with a single guanosine, adopt a dimeric structure that sequesters the 5′-cap and exposes high-affinity NC binding sites, whereas 5′-capped transcripts that begin with two (less common) or three guanosines, form monomers that are substantially remodeled, compared to the dimeric RNA. The energetics are exquisitely balanced, with remodeling occurring at the energetic cost of a single G-C base pair (~3 kCal/mol).

**Figure 20:**
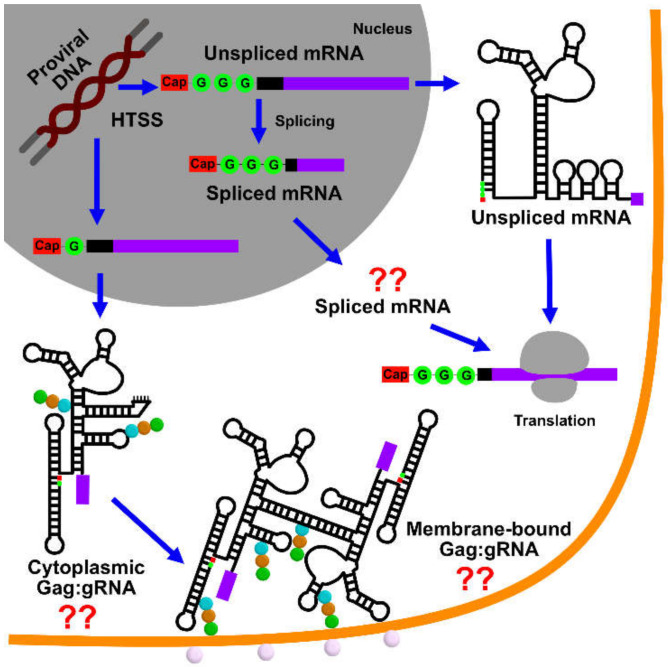
Open questions regarding the structural biology of HIV-1 genome packaging. Structures of spliced mRNAs that avoid packaging are unknown, and structures of gRNA-Gag complexes that anchor the genome to the plasma membrane and nucleate virus assembly are also unknown.

Future studies of retroviral packaging mechanisms will benefit from studies of ever larger RNAs and protein–RNA complexes, including spliced RNA transcripts and unspliced transcripts bound to Gag, both within the cytoplasm and associated with the plasma membrane; Figure 20. Confocal imaging studies indicate that the ribonucleoprotein complex targeted to the plasma membrane assembly sites comprise about a dozen Gag proteins bound to the RNA genome, and this complex is too large to study using the presently available solution-based NMR methods. Solution-state NMR studies of larger complexes would require the development of improved methodologies for enhancing sensitivity and resolution, in addition to the development of improved labeling methods for rapid and economical preparation of segment- and site-specifically labeled RNAs. In this regard, Wang et al. developed a method for site-specific isotopic labeling of RNAs [410], but this potentially powerful technology requires specialized equipment and is yet to be widely adopted. Expanded use of ^1^H-^15^N correlated NMR methods for detecting the H2 protons of ^15^N-labeled adenosines [411] (e.g., for detection of pseudo-contact shifts in RNAs with site-specifically bound proteins that contain paramagnetic tags) should prove useful for studies of larger systems. The continued development of hybrid approaches that combine high-resolution, short-range NMR restraints, with lower resolution global structural restraints provided by cryo-EM or SAXS, could lead to the derivation of more accurate structures for systems of greater size and complexity. In addition, solid-state NMR methods could prove useful for studying large protein-RNA assemblies, if appropriately homogeneous samples can be prepared.

In summary, NMR contributed considerably to our understanding of the atomic-level structural determinants of retroviral genome selection and packaging, more so than any other structural methodology. Since the first reported NMR structure of a retroviral zinc knuckle 30 years ago, all 3D structural information for retroviral zinc knuckle domains, NC proteins, and NC–RNA complexes were provided by NMR. Challenges associated with the crystallization of structurally flexible polycationic proteins such as NC, or structurally heterogeneous RNA polyanions, makes NMR an ideal methodology for studying retroviral RNA packaging. The NMR techniques developed over the course of these studies should be broadly applicable to the rapidly expanding field of non-coding cellular RNAs. As questions about genome packaging shift to larger systems, new approaches involving hybrid methodologies, advancements in labeling techniques, and possibly solid-state NMR will likely make important future contributions.
