# Correction: Boyd, P.S., et al. NMR Studies of Retroviral Genome Packaging. *Viruses* **2020**, *12*, 1115

**DOI:** 10.3390/v13030453

**Published:** 2021-03-11

**Authors:** Patricia S. Boyd, Janae B. Brown, Joshua D. Brown, Jonathan Catazaro, Issac Chaudry, Pengfei Ding, Xinmei Dong, Jan Marchant, Colin T. O’Hern, Karndeep Singh, Canessa Swanson, Michael F. Summers, Saif Yasin

**Affiliations:** Howard Hughes Medical Institute, Department of Chemistry and Biochemistry, University of Maryland Baltimore County, Baltimore, MD 21250, USA; pboyd2@umbc.edu (P.S.B.); janaeb@umbc.edu (J.B.B.); jdbrown@umbc.edu (J.D.B.); jcataz@umbc.edu (J.C.); ichaudr1@umbc.edu (I.C.); dingp@umbc.edu (P.D.); dx1@umbc.edu (X.D.); janm@umbc.edu (J.M.); cohern1@umbc.edu (C.T.O.); ksingh5@umbc.edu (K.S.); canessa@umbc.edu (C.S.); saif.yasin@som.umaryland.edu (S.Y.)

**Text Correction**

There was an error in the original article [1]. The wording suggested that conclusions of some studies were corrected in subsequent research, when in fact the mentioned studies focused on two different viruses with different behaviors. A correction has been made to the Introduction section, paragraph 3, sentence 3, and subsequent references have been renumbered:

Dimerization of the MoMuLV genome occurs co-transcriptionally or near the site of the provirus [53–57] whereas HIV genomes dimerize randomly [54,58], most likely after being exported from the nucleus [59] and possibly not until they reach the plasma membrane [30].

The authors apologize for any inconvenience caused and state that the scientific conclusions are unaffected. The original article has been updated.

**References Added**

54 Onafuwa, A.; An, W.; Robson, N.D.; Telesnitsky, A. Human immunodeficiency virus Type 1 genetic recombination is more frequent than that of Moloney Murine Leukemia Virus despite similar template switching rates. *J. Virol*. **2003**, *77*, 4577–4587.
55 Flynn, J.A.; King, S.R.; Telesnitsky, A. Nonrandom dimerization of murine leukemia virus genomic RNAs. *J. Virol.* **2004**, *78*, 12129–12139.
56 Kharytonchyk, S.A.; Kireyeva, A.I.; Osipovich, A.B.; Fomin, I.K. Evidence for preferential copackaging of Moloney murine leukemia virus genomic RNAs transcribed in the same chromosomal site. *Retrovirology* **2005**, *2*, doi:10.1186/1742-4690-2-3.
57 Rasmussen, S.V.; Pedersen, F.S. Co-localization of gammaretroviral RNAs at their transcription site favours co-packaging. *J. Gen. Virol.* **2006**, *87*, 2279–2289.
58 Rhodes, T.; Wargo, H.; Hu, W.-S. High rates of human immunodeficiency virus type 1 recombination: Near-random segregation of markers one kilobase apart in one round of viral replication. *J. Virol.* **2003**, *77*, 11193–11200.
